# Preclinical Evidence of *Curcuma longa* Linn. as a Functional Food in the Management of Metabolic Syndrome: A Systematic Review and Meta-Analysis of Rodent Studies

**DOI:** 10.3390/biomedicines13081911

**Published:** 2025-08-05

**Authors:** Samuel Abiodun Kehinde, Zahid Naeem Qaisrani, Rinrada Pattanayaiying, Wai Phyo Lin, Bo Bo Lay, Khin Yadanar Phyo, Myat Mon San, Nurulhusna Awaeloh, Sasithon Aunsorn, Ran Kitkangplu, Sasitorn Chusri

**Affiliations:** 1 Biomedical Technology Research Group for Vulnerable Populations and School of Health Science, Mae Fah Luang University, Chiang Rai 57100, Thailand; samuelabiodun.research@mfu.ac.th (S.A.K.); qaisrani.research@mfu.ac.th (Z.N.Q.); 6431804208@lamduan.mfu.ac.th (W.P.L.); 6531804115@lamduan.mfu.ac.th (K.Y.P.); 6531804120@lamduan.mfu.ac.th (M.M.S.); 6551811005@lamduan.mfu.ac.th (S.A.); 6451811006@lamduan.mfu.ac.th (R.K.); 2 Biochemical/EnTox Lab., Faculty of Basic Medical Sciences, Ajayi Crowther University, Oyo 211001, Nigeria; 3 Department of Chemical Engineering, Faculty of Engineering & Architecture, Balochistan University of Information Technology, Engineering and Management Sciences (BUITEMS), Quetta 87300, Pakistan; 4 Department of Food Innovation and Professional Chef, Faculty of Science and Technology, Suan Sunandha Rajabhat University, Bangkok 10300, Thailand; rinrada.pa@ssru.ac.th; 5 School of Information Technology, Mae Fah Luang University, Chiang Rai 57100, Thailand; 6431306113@lamduan.mfu.ac.th; 6 Thai Faculty of Allied Health Science, Nakhon Ratchasima College, Nakhon Ratchasima 30000, Thailand; nurulhusna@nmc.ac.th

**Keywords:** *Curcuma longa*, inflammatory markers, metabolic syndrome, oxidative stress, preclinical evidence, systematic review, meta-analysis

## Abstract

**Background/Objectives**: Metabolic syndrome (MetS) is a multifactorial condition characterized by abdominal obesity, dyslipidemia, insulin resistance, hypertension, and chronic inflammation. As its global prevalence rises, there is increasing interest in natural, multi-targeted approaches to manage MetS. *Curcuma longa* Linn. (turmeric), especially its active compound curcumin, has shown therapeutic promise in preclinical studies. This systematic review and meta-analysis evaluated the effects of *Curcuma longa* and its derivatives on MetS-related outcomes in rodent models. **Methods**: A comprehensive search was conducted across six databases (PubMed, Scopus, AMED, LILACS, MDPI, and Google Scholar), yielding 47 eligible in vivo studies. Data were extracted on key metabolic, inflammatory, and oxidative stress markers and analyzed using random-effects models. Results were presented as mean differences (MD) with 95% confidence intervals (CI). **Results**: Meta-analysis showed that curcumin significantly reduced body weight (rats: MD = −42.10; mice: MD = −2.91), blood glucose (rats: MD = −55.59; mice: MD = −28.69), triglycerides (rats: MD = −70.17; mice: MD = −24.57), total cholesterol (rats: MD = −35.77; mice: MD = −52.61), and LDL cholesterol (rats: MD = −69.34; mice: MD = −42.93). HDL cholesterol increased significantly in rats but not in mice. Inflammatory cytokines were markedly reduced, while oxidative stress improved via decreased malondialdehyde (MDA) and elevated superoxide dismutase (SOD) and catalase (CAT) levels. Heterogeneity was moderate to high, primarily due to variations in curcumin dosage (ranging from 10 to 500 mg/kg) and treatment duration (2 to 16 weeks) across studies. **Conclusions**: This preclinical evidence supports *Curcuma longa* as a promising functional food component for preventing and managing MetS. Its multi-faceted effects warrant further clinical studies to validate its translational potential.

## 1. Introduction

Metabolic syndrome (MetS) is a complex and multifactorial disorder characterized by a cluster of interrelated metabolic abnormalities that significantly increase the risk of cardiovascular disease (CVD), type 2 diabetes mellitus (T2DM), and all-cause mortality. These abnormalities commonly include central obesity, insulin resistance, elevated blood pressure, hyperglycemia, dyslipidemia (notably elevated triglycerides and reduced levels of high-density lipoprotein cholesterol), and systemic inflammation [1]. The rising global prevalence of MetS, especially in low- and middle-income countries, has become a major public health concern. This increase has been largely attributed to sedentary lifestyles, unhealthy dietary patterns, urbanization, and, to a lesser extent, genetic predispositions [2]. Recent studies report that the global prevalence of MetS among adults is approximately 25%, with some regions, such as the Middle East and South Asia, exceeding 30% [3]. Beyond prevalence, MetS is associated with a significantly increased risk of all-cause mortality and cardiovascular events, with pooled hazard ratios of 1.6 and 2.3, respectively, compared to individuals without MetS [4,5]. These findings highlight the critical need for effective, multi-targeted interventions, reinforcing the value of preclinical research in guiding therapeutic development.

Given its clinical complexity and growing impact on global health, there is a pressing need for effective preventive and therapeutic strategies to manage MetS. While pharmacological treatments are available, they often target individual components of MetS and may be associated with long-term side effects. In recent years, there has been a surge of interest in natural products and dietary interventions due to their broad-spectrum bioactivities, safety profiles, and holistic benefits. One such natural compound is curcumin, the principal curcuminoid derived from the rhizome of *Curcuma longa* (turmeric), a plant widely used in traditional medicine systems, such as Ayurveda and Thai and Traditional Chinese Medicine [6].

Curcumin has been extensively studied for its wide range of pharmacological properties, including anti-inflammatory, antioxidant, lipid-lowering, anti-obesity, and insulin-sensitizing effects [7]. These multi-faceted activities suggest their potential in targeting multiple pathophysiological pathways involved in MetS. Mechanistically, curcumin is known to modulate various molecular targets, including inflammatory cytokines (e.g., TNF-α), transcription factors (e.g., NF-κB), enzymes (e.g., cyclooxygenase-2), and markers of oxidative stress. Additionally, it helps restore metabolic homeostasis by improving insulin sensitivity, regulating lipid metabolism, and reducing adipogenesis and visceral fat accumulation [8].

The underlying pathophysiology of MetS is driven primarily by chronic low-grade inflammation, oxidative stress, and adipose tissue dysfunction. Central to its progression are altered cellular signaling pathways, such as the insulin/PI3K/Akt pathway, which regulates glucose uptake and lipid metabolism and is commonly impaired in insulin resistance. Dysregulation of AMP-activated protein kinase (AMPK) signaling, an energy sensor that controls metabolic homeostasis, also plays a pivotal role, as its inactivation contributes to lipid accumulation, mitochondrial dysfunction, and inflammation in key metabolic tissues, such as the liver, muscle, and adipose tissue [9,10]. Additionally, the nuclear factor-kappa B (NF-κB) signaling pathway has been implicated in the inflammatory cascade underlying MetS. Its activation leads to increased transcription of pro-inflammatory cytokines (e.g., TNF-α, IL-6) that disrupt insulin signaling and exacerbate metabolic derangements. The crosstalk between NF-κB and c-Jun N-terminal kinase (JNK) pathways further amplifies insulin resistance, particularly through phosphorylation of insulin receptor substrate-1 (IRS-1), thereby impairing insulin signal transduction [11]. These interconnected molecular events establish a pathogenic network that perpetuates MetS progression and makes targeting these pathways a promising strategy in both pharmacologic and nutritional interventions.

Animal models, particularly rodent models, have served as valuable tools in exploring the pathogenesis of MetS and the therapeutic potential of curcumin. A variety of rodent species and strains, such as Wistar rats, Sprague Dawley rats, and genetically modified mice, have been employed to mimic the human condition of MetS through diverse induction methods. These include high-fat diets (HFD), high-fructose diets, genetic manipulation, or a combination of dietary and hormonal alterations [12,13]. Numerous preclinical studies have investigated the effects of curcumin in these models. For instance, Nabiuni et al. [14] assessed its efficacy in Wistar rats with polycystic ovary syndrome (PCOS)-induced MetS, while Wu et al. [15] used a high-fat diet to induce MetS in Sprague Dawley rats, administering curcumin at varying doses. Similarly, Chiu et al. [16] explored the effects of tetrahydrocurcumin (THC), a more bioavailable curcumin metabolite, in C57BL/6J mice, demonstrating improvements in insulin sensitivity and reductions in adiposity over a 10-week period.

These studies differ in their methodologies, ranging in curcumin dosage (typically between 20 and 100 mg/kg), routes of administration (primarily oral), and treatment durations (ranging from 2 to 10 weeks). Despite these variations, many studies consistently report curcumin’s beneficial effects on glucose regulation, lipid profiles, blood pressure, weight control, and inflammatory markers. Importantly, several investigations have also examined curcumin’s ability to counter oxidative stress and chronic inflammation, both of which are key drivers of MetS pathology [17,18]. Moreover, curcumin’s potential synergistic interactions with other bioactive agents and lifestyle interventions have been highlighted, broadening its scope as part of integrative approaches to MetS management.

This study aims to critically evaluate the preclinical evidence on the efficacy of *Curcuma longa* in modulating parameters of MetS, with implications for functional food development, as the systematic review and meta-analysis attempt to critically evaluate and synthesize preclinical evidence on the therapeutic efficacy of curcumin in rodent models of metabolic syndrome. By consolidating data across various studies, this review seeks to determine the consistency of outcomes, explore dose–response relationships, highlight potential mechanisms of action, and assess the quality and translational value of existing animal studies. Ultimately, this work will help inform future research directions and support the development of curcumin-based interventions for the prevention and management of MetS in humans.

## 2. Materials and Methods

**Figure 1:**
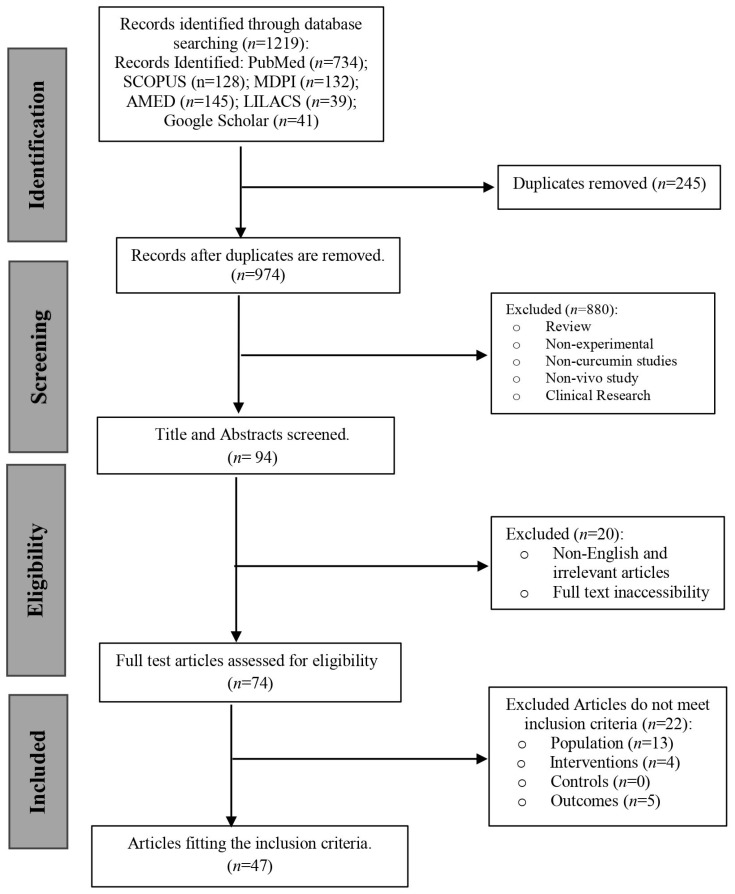
PRISMA flow diagram of the systematic review process.

This systematic review was conducted in accordance with the Preferred Reporting Items for Systematic Reviews and Meta-Analyses (PRISMA) guidelines [19]. Figure 1 displays the PRISMA Protocols checklist. The systematic review was registered priori hoc on the International Platform of Registered Systematic Review and Meta-Analysis Protocols (INPLASY^®^) under the identifier INPLASY2024110032 (https://inplasy.com/inplasy-2024-11-0032/, accessed on 6 November 2024). A meta-analysis of extracted data focused on parameters related to metabolic syndrome, including body weight gain, lipid profiles (total cholesterol, HDL cholesterol, LDL cholesterol), triglycerides, blood glucose, and insulin levels, was conducted using MetaAnalysisOnline.com (https://metaanalysisonline.com; accessed on 17 June 2025) [20].

### 2.1. Search Strategy

Databases including PubMed, Scopus, AMED, LILACS, Google Scholar, and MDPI were searched without temporal limitations. A variety of descriptors, including grouped Boolean operators, such as (“Curcumin” OR “*Curcuma longa*” OR “Diferuloylmethane”) AND (“in vivo”) AND (“rat” OR “mice”) AND (“metabolic syndrome” OR “insulin resistance” OR “type 2 diabetes”), were employed to identify pertinent studies. The Boolean operators “AND” and “OR” were used to combine the descriptors. The search strategy is outlined in the Supplementary Materials (Supplementary Table S1). The review was limited to papers published on original and experimental studies in rats and mice. All of the articles retrieved through these searches were transferred to Microsoft Excel 19, and duplicates were removed. Initial screening was conducted through title and abstract review, resulting in the removal of unrelated articles. Afterwards, the remaining articles underwent a critical evaluation by screening the full text. Articles without available full texts or those deemed irrelevant were also excluded.

### 2.2. Eligibility Criteria

The research question for the current study was the following: “What are the mechanisms of curcumin and turmeric extract in preventing the onset and progression of metabolic syndrome as evidenced by in vivo studies?” An international database of prospectively recorded systematic reviews in health and social care, along with the PICO (Population, Intervention, Comparator, and Outcomes) components (Table 1), formed the basis for the inclusion and exclusion criteria. Two independent researchers assessed the eligibility requirements. The titles and abstracts of each article served as the foundation for the preliminary evaluation. This study included studies published in English with full texts, excluding duplicate papers. Human studies, in vitro studies, editorials, case reports, dissertations, reports, theses, and papers not pertinent to the primary issues were also removed.

### 2.3. Information Sources

A comprehensive literature search of electronic databases was conducted, including the US National Library of Medicine and the National Institutes of Health, PubMed (http://www.pubmed.gov, accessed on 4 October 2024), SCOPUS (http://www.scopus.com, accessed on 2 October 2024), the Allied and Complementary Medicine Database (AMED), AMED (https://www.ebsco.com/products/research-databases, accessed on 3 October 2024), the Latin American and Caribbean Health Sciences Literature, LILACS (https://lilacs.bvsalud.org, accessed on 4 October 2024), the Multidisciplinary Digital Publishing Institute, MDPI (https://www.mdpi.com, accessed on 4 October 2024), and Google Scholar (https://scholar.google.com/, accessed on 4 October 2024). These databases were searched for findings published until November, 2024 (Supplementary Table S1).

### 2.4. Data Retrieval and Synthesis

Two independent reviewers screened titles and abstracts to identify eligible studies. In cases of disagreement, a third reviewer resolved the conflict by determining whether the study met the inclusion criteria. To minimize subjectivity during data collection and entry, three reviewers independently extracted data from the included studies and recorded them in separate databases. Data were abstracted using standardized forms that captured key study characteristics, including the first author’s name, publication year, publication country, sample size, animal gender, age, and strain, methodology, intervention strategies, treatment durations, significant outcomes, and principal conclusions. Additionally, indices, such as environmental/feeding conditions, type of treatment, induction methods, dose, and duration, were also expressed. When effect sizes could not be extracted or calculated from the published data, corresponding authors were contacted via email for additional statistical information. Finally, the databases were cross-checked, and any discrepancies were resolved through discussion among the reviewers. To enhance the results’ visual representation, the data were arranged and depicted with figures, and some were displayed as tables.

### 2.5. Quality Assessment

The quality of evidence from the included studies was assessed using the Collaborative Approach to Meta-Analysis and Review of Animal Data from Experimental Studies (CAMRADES) framework, designed to improve the design, execution, and reporting of preclinical studies in systematic reviews and meta-analyses [21]. This evaluation examined various factors, including the risk of bias in individual studies, the directness of the evidence, the precision of effect estimates, heterogeneity among studies, and the potential for publication bias. Bias, in this context, refers to systematic deviations from accurate findings or inferences that can distort study results. A structured checklist, created by the Systematic Review Centre for Laboratory Animal Experimentation (SYRCLE), was used to evaluate the risk of bias. This instrument, derived from the Cochrane Collaboration’s Risk of Bias Tool [22,23], comprises ten items categorized into six primary domains: selection bias, performance bias, detection bias, attrition bias, reporting bias, and other sources of bias. Each domain was evaluated using one of three responses: “Yes” (indicating low risk of bias), “No” (indicating high risk), or “NC” (not clear, due to insufficient information). A point was allocated for each item evaluated as “Yes.” Two reviewers independently evaluated each study and classified the risk of bias as “low,” “unclear,” or “high” in the following domains: sequence generation, baseline characteristics, allocation concealment (selection bias), random housing and blinding (performance bias), random outcome assessment and blinding (detection bias), incomplete outcome data (attrition bias), selective outcome reporting (reporting bias), and other potential sources of bias. Upon concluding their assessments, the reviewers engaged in a comparative analysis and discussion of their findings. Discrepancies in scoring were addressed through consultation with a third reviewer to achieve consensus.

### 2.6. Statistical Analysis

A statistical analysis was performed using an online tool, MetaAnalysisOnline.com (https://metaanalysisonline.com; accessed on 17 June 2025) [20]. The impact of curcumin on metabolic indices and lipid profile was investigated with a corresponding CI of 95% and expressed as the mean difference (MD) when compared to the controls. The mean values, standard deviations (SD), and sample sizes from the intervention and control groups were promptly imported into the application. Standard errors (SE) given in studies were transformed into standard deviations (SD) before data entry. Continuous outcomes were analyzed using a random effects model, owing to the expected methodological and biological heterogeneity among studies. The mean difference (MD) with a 95% confidence interval (CI) was used to determine effect sizes, considering the different measurement scales employed in the research. The variation between studies was determined using the method of moments (DerSimonian and Laird), with heterogeneity evaluated through Cochran’s Q-test and quantified using the I^2^ statistic. An I^2^ value beyond 50% indicated considerable heterogeneity. Publication bias was assessed visually using funnel plots created using MetaAnalysisOnline.com.

## 3. Results

### 3.1. Study Selection

The process of study selection is represented in the PRISMA flow diagram (Figure 1). A total of 1219 records were first identified using electronic database searches, comprising contributions from PubMed (*n* = 734), SCOPUS (*n* = 128), AMED (*n* = 145), LILACS (*n* = 39), MDPI (*n* = 132), and Google Scholar (*n* = 41). Following the elimination of 245 duplicate records, 974 distinct records were retained for screening. In the title and abstract screening step, 880 records were rejected for failing to meet the inclusion criteria. The exclusions were mainly attributed to the articles being reviews, non-experimental studies, unconnected to curcumin, non-in vivo investigations, clinical research, non-English publications, or otherwise inappropriate to the topic under investigation. This resulted in 94 records for additional evaluation. Twenty items were eliminated after a comprehensive assessment of the whole text due to inaccessibility or classification as reviews or analytical articles. Seventy-four full-text papers were evaluated for eligibility. After a comprehensive evaluation, 22 additional publications were removed for failing to meet the specified inclusion criteria, as 13 did not have the required population, 4 did not satisfy the intervention criteria, and 5 lacked outcome data. None were excluded based on control criteria.

A total of 47 studies [24,25,26,27,28,29,30,31,32,33,34,35,36,37,38,39,40,41,42,43,44,45,46,47,48,49,50,51,52,53,54,55,56,57,58,59,60,61,62,63,64,65,66,67,68,69,70] met all of the established inclusion criteria and were included in the final systematic review. Statistical data for these studies were obtained either directly from published publications or by contacting the corresponding authors by email. Nevertheless, for specific research, the necessary data could not be acquired due to the authors’ lack of responsiveness. Consequently, articles with inadequate data [25,26,27,28,35,40,41,42,49,53,60,61,64,66,69] for meta-analysis were exclusively incorporated into the qualitative segment of the systematic review. The very first study was published in 1970, whereas 82.98% of the studies were published in the last decade (2014–2024), demonstrating a growing interest in the efficacy of *Curcuma longa* Linn as a functional food.

### 3.2. Risk of Bias Assessment

The SYRCLE tool was used across the included studies to assess the risk of bias, revealing different bias levels with significant concerns in several domains. Selection bias was high in several studies, indicating that randomization and allocation concealment were poorly executed or inadequately reported. Most studies had a low risk of baseline group equivalence, suggesting that appropriate adjustments were made or that the groups were intrinsically comparable. Performance bias showed a mix of low and unclear risks, implying that many studies did not adequately report animal housing conditions. However, blinding of caregivers and investigators was generally well-handled, with most studies rated as low risk, reducing the influence of observer bias. Detection bias was also consistently rated as low, indicating that outcome assessors were usually blinded, which strengthens the validity of the results. Attrition bias, on the other hand, was often rated as unclear or low, suggesting that many studies failed to account for all animals, which could potentially skew the outcomes. Reporting bias was a significant concern, with many studies receiving a “High” risk rating, suggesting selective reporting of results. Additionally, “Other sources of bias” were frequently rated as high, showing potential methodological conflicts of interest.

**Figure 2:**
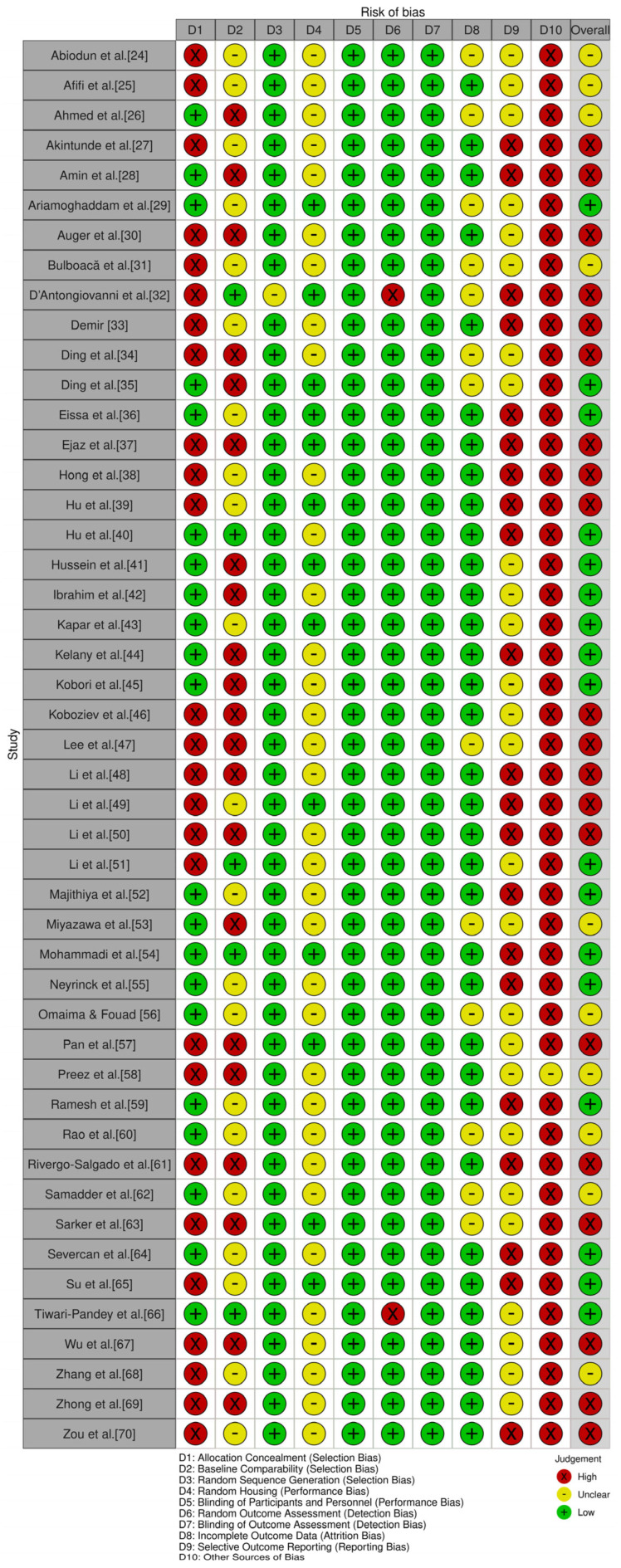

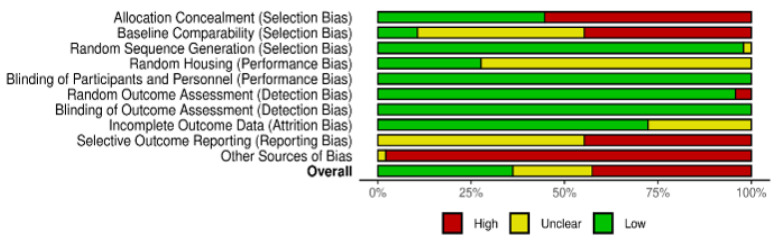
Quality of reporting and bias evaluation conducted with SYRCLE’s risk of bias tool. The upper panel illustrates the quality of reporting and bias risk in the included studies, while the lower panel evaluates biases related to selection, performance, detection, attrition, and other factors [24,25,26,27,28,29,30,31,32,33,34,35,36,37,38,39,40,41,42,43,44,45,46,47,48,49,50,51,52,53,54,55,56,57,58,59,60,61,62,63,64,65,66,67,68,69,70,71].

The risk of bias in the included studies is shown in Figure 2 (generated from Supplementary Tables S2 and S3), which illustrates the quality of reporting and bias assessment conducted using SYRCLE’s risk of bias tool to evaluate biases related to selection, performance, detection, attrition, and other factors. Twenty studies [24,28,30,32,33,34,37,38,39,46,47,48,50,51,57,59,63,67,69,70] had overall high biases, seventeen studies [29,35,36,40,41,42,43,44,45,49,52,54,55,59,64,65,66] had overall low risk of bias, and ten studies [24,25,26,31,53,56,58,60,62,68] had a low risk of bias. Although some studies effectively minimized certain biases (i.e., performance and detection biases), there are several issues with selection bias, attrition bias, and especially reporting bias that undermine the reliability of the findings. Therefore, a meta-analysis was performed for further clarification.

### 3.3. Study Characteristics

**Figure 3:**
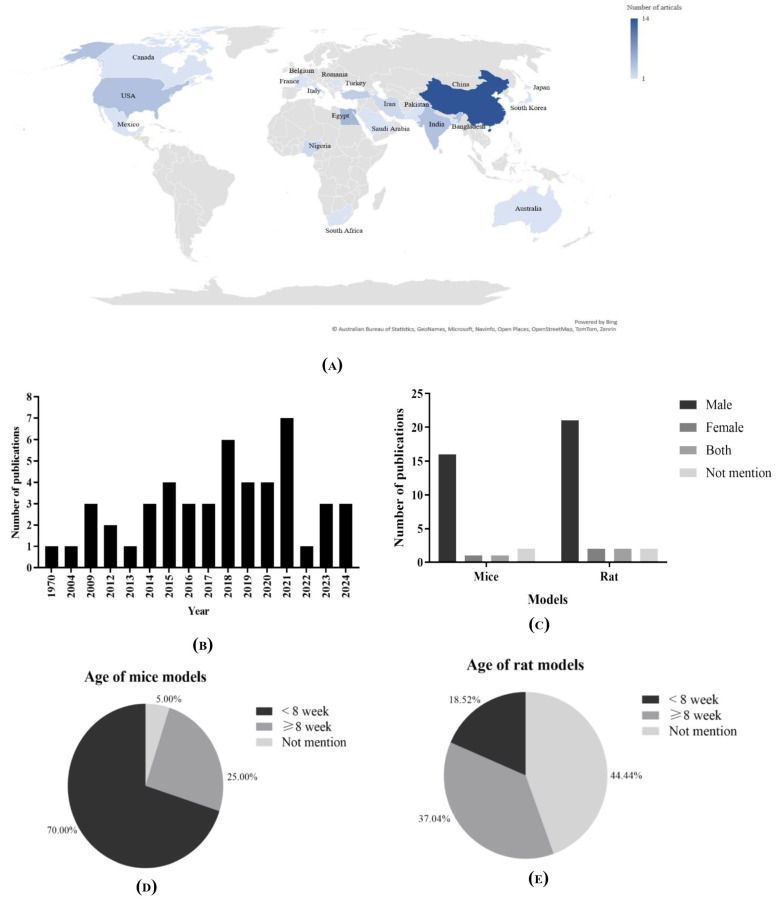
Geographical distribution (**A**), publication year (**B**), gender (**C**), and age (**D**,**E**) distribution of the rodents used in the included studies reflect the demographics of rodents assessed for the beneficial effects of curcumin/*Curcuma longa* extracts in research on metabolic syndrome.

The general characteristics of the included studies are presented in Table 2 and Figure 3.

#### 3.3.1. Demographic Data

The studies were conducted in 20 countries, with China as a leading country with 29.78% of studies (*n* = 14) [34,35,38,39,40,48,49,50,51,57,65,68,69,70], as well as Egypt (*n* = 5) [25,36,41,44,56], the USA (*n* = 4) [37,46,53,67], India [52,59,60,62], Turkey (*n* = 3) [33,43,44], Nigeria (*n* = 2) [24,27], Iran [29,54], and 1 study from Pakistan, Canada, Saudi Arabia, South Africa, Bangladesh, France, Korea, Mexico, Romania, Japan, Australia, and Belgium [26,28,30,31,42,45,47,55,58,61,63,66], respectively. The selected studies were published between 2004 and 2024, except for one study in 1970 [60]. The years 2015, 2018, 2019, 2020, and 2021 have four or more publications each.

#### 3.3.2. Animal Models of Included Studies

As depicted also in Table 2 and Figure 3, the selected studies (*n* = 47) used various rodent species, such as Wistar rats, Sprague Dawley rats, and C57BL/6J mice. Rats were the leading species (*n* = 28) with 59.57% [24,25,26,27,28,29,31,32,33,34,36,39,40,41,42,43,44,49,50,54,56,58,59,60,61,64,65,68], while the remaining 40.43% (*n* = 19) were mice [30,35,37,38,45,46,47,49,51,52,53,55,57,62,63,66,67,69,70]. A closer look at the age of animal models used in the included studies reveals notable differences between rat and mouse experiments. Among the rat studies, 39.28% (*n* = 11) explicitly stated the use of rats aged 8 weeks or older [26,29,33,34,36,39,40,42,43,49,51,56]. In contrast, only 17.29% (*n* = 4) used rats younger than 8 weeks [27,32,60,61].

Interestingly, a substantial portion, 44.44% (*n* = 12), did not specify the age of the rats used, which introduces uncertainty regarding developmental stage and metabolic maturity [24,25,28,31,41,44,54,56,59,64,65,68]. In contrast, mouse models (*n* = 19) showed a clearer trend toward using younger animals. A majority, 68.42% (*n* = 13), used mice under 8 weeks of age [30,35,37,39,45,46,48,51,55,57,62,63,67], while 21.05% (*n* = 4) used mice aged 8 weeks or older [47,53,69,70]. Only 10.53% (*n* = 2) of the mouse studies failed to mention the age [52,66].

The classification of rodents age into “<8 weeks” and “≥8 weeks” reflects important developmental milestones, with rodents under 8 weeks considered juvenile or peripubertal, experiencing ongoing growth and hormonal changes, while rats 8 weeks or older are regarded as young adults with mature physiology, making them more suitable for toxicological, pharmacological, and disease modeling studies; this distinction aligns with OECD and NIH guidelines to ensure consistency, reduce variability, enhance translational validity, and uphold ethical standards in biomedical research. These age-related differences are important to consider, as developmental stage can significantly influence physiological responses, metabolism, and the outcomes of curcumin intervention. The lack of age specification in a notable number of studies highlights the need for better reporting standards in preclinical research.

Among the 19 studies that utilized mouse models, the majority (78.95%, *n* = 15) exclusively used male mice, as reported in studies by Ding et al. [35], Ejaz et al. [37], Hong et al. [38], Kobori et al. [45], Koboziew et al. [46], Lee et al. [47], Li et al. [49,51], Majithiya et al. [62], Miyazawa et al. [53], Neyrinck et al. [55], Pan et al. [57], Wu et al. [67], Zhong et al. [69], and Zou et al. [70]. Only one study (5.26%) employed female mice exclusively [30], while another [66] included both male and female subjects, also representing 5.26%. Notably, 10.53% of the mouse studies (*n* = 2) did not specify the sex of the animals used [62,63]. Similarly, in the 28 studies that used rat models, 78.57% (*n* = 22) focused solely on male rats. This was evident in work by Abiodun et al. [24], Afifi et al. [25], Ahmed et al. [26], Akintunde et al. [27], Bulboaca et al. [31], D’Antongiovanni et al. [32], Ding et al. [34], Eissa et al. [36], Hu et al. [39,43], Hussein et al. [30], Kapar et al. [43], Kelany et al. [44], Li et al. [49,50], Omaima and Fouad [56], Preez et al. [58], Ramesh et al. [59], Rivego-Sagado et al. [61], Severcan et al. [64], Su et al. [65], and Zhang et al. [68]. Only two studies (7.14%) by Mohammadi et al. [54] and Rao et al. [60] used only female rats. Two additional studies (7.14%) included both sexes [28,42], while the remaining two (7.14%) did not report the sex of the animals [29,33]. This trend reveals a notable sex bias toward the use of male animals in preclinical studies, which may limit the generalizability of findings and underscores the need for more sex-balanced research designs.

Environmental and feeding conditions (Supplementary Table S5) were variably reported across studies. Temperature was specified in 51.11% (*n* = 24) of the included studies, with values generally ranging between 21 °C and 26 °C [26,27,28,31,32,33,41,43,44,45,46,47,48,49,50,51,52,57,58,61,62,64,69,70]. A consistent 12:12 h light–dark cycle was described in 61.7% (*n* = 29) of studies [26,27,30,31,32,33,34,35,43,44,45,47,48,49,50,51,52,53,55,57,58,59,61,62,63,64,68,69,70]. Humidity levels were reported in only 23.40% (*n* = 11) of studies, with most values falling within the 50–60% range [26,31,32,33,41,43,45,48,57,61,62]. As for feeding, ad libitum administration was the most commonly used approach, reported in 46.67% (*n* = 21) of the studies [27,28,30,31,36,41,43,44,45,50,51,52,55,57,58,59,61,62,63,66,69]. However, 27.66% (*n* = 13) of the studies did not report any details about environmental conditions or feeding schedules [24,25,29,37,38,39,40,42,56,60,63,65,67], making it difficult to assess the degree of environmental control or its potential influence on outcomes.

### 3.4. Induction Method of Metabolic Syndrome in Animal Models

**Figure 4:**
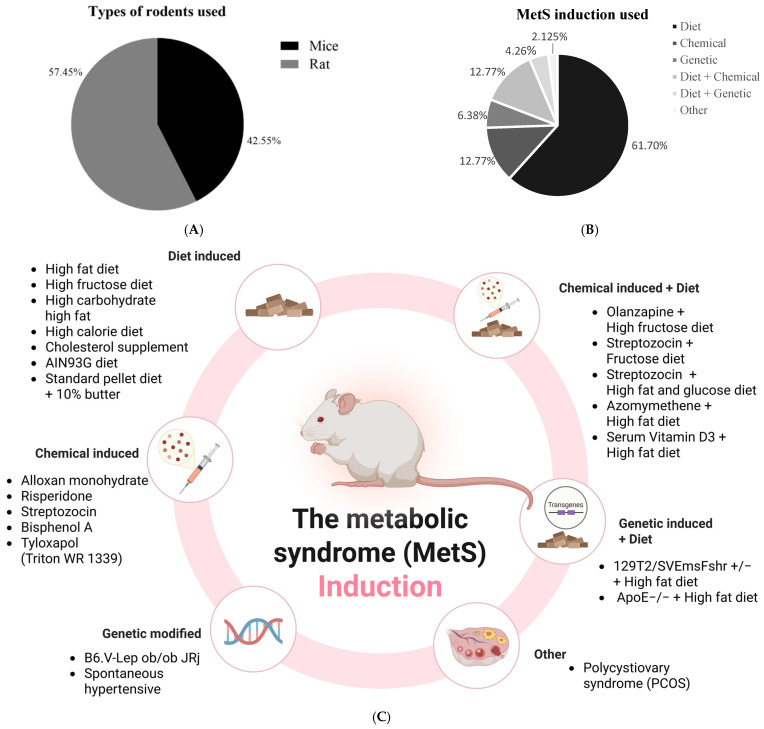
Types of rodents used in the included studies (**A**) and the detailed methods (**B**,**C**) for inducing metabolic syndrome in these rodents.

In the reviewed studies, six (6) primary approaches to metabolic syndrome induction were identified: chemical, genetic, dietary, combined diet–genetic, combined diet–chemical-induced, and others (Figure 4 and Supplementary Table S6). These methods reflect the multifactorial nature of metabolic syndrome and efforts to accurately model its pathophysiology in an experimental setting.

#### 3.4.1. Dietary Induction of Metabolic Syndrome Models

Dietary induction was the most frequently employed method for modeling metabolic syndrome, accounting for 61.70% (*n* = 29) of all included studies [24,26,28,29,32,34,35,36,37,39,41,42,43,44,45,46,47,48,50,53,57,58,60,61,63,64,68,69]. This approach involves feeding a high-fat diet [24,32,35,39,41,44,46,47,48,50,53,57,69], a high-fructose diet [25,26,28,29,34,42,43,68], a high-carbohydrate–high-fat diet [36,58], a high-calorie diet [61], a cholesterol supplement [60], an AIN93G diet [45], and a standard pellet diet + 10% butter [63]. The use of a high-fat diet (*n* = 14) accounted for the majority (48.27%) of dietary induction of metabolic syndrome. This approach is very common, and it provides an important link between lifestyle factors and metabolic syndrome risk.

#### 3.4.2. Chemical Induction of Metabolic Syndrome

Chemical induction was the second most employed method for modeling metabolic syndrome, accounting for 12.77% (*n* = 6) of all included studies [27,30,33,38,52,62]. This approach involves the administration of toxic agents to replicate key pathological features of metabolic syndrome, such as oxidative stress, insulin resistance, central obesity, dyslipidemia, hypertension, inflammation, and non-alcoholic fatty liver disease. These models are favored due to their cost-effectiveness, ease of administration, and relatively rapid onset of pathological deficits, making them both reliable and reproducible. Among the chemical inducers, Bisphenol A (BPA) was the most commonly used (*n* = 2, 33.33%) agent. It was featured in two studies [27,38] to induce the pathologies associated with metabolic syndrome. Three studies (*n* = 3) used STZ to chemically induce metabolic disturbances, either alone or in combination with dietary models, such as HFD or fructose, to simulate insulin resistance and hyperglycemia characteristic of metabolic syndrome [31,33,56]. Other used chemical agents include Risperidone [30], Triton WR1339 [52], and Alloxan Monohydrate [62]. Collectively, these agents offer diverse and mechanistically relevant models for studying metabolic syndrome pathologies.

#### 3.4.3. Genetic Induction

Three (3) studies (6.38%) employed transgenic mouse models to investigate genetically predisposed forms of metabolic syndrome. Two of the three studies used spontaneous hypertensive rats (SHRs) to mimic metabolic syndrome [40,49], while ob/ob mice (B6.V-Lepob/ob/JRj) were used in only one study [55].

#### 3.4.4. Combined Diet and Genetic Induction of Metabolic Syndrome Model

A further 4.26% (*n* = 2) of the studies employed a combined dietary and genetic approach. The approaches of Tiwari-Pandey et al. [66] and Zou et al. [70] utilized a high-fat diet in conjunction with genetic modifications via 129T2/SVEmFshr +/− and ApoE−/−+. Although underutilized, this combined method also offers a holistic model of metabolic syndrome disease development.

#### 3.4.5. Combined Diet and Chemical Induction of Metabolic Syndrome Model

Also, 8.51% of the studies (*n* = 5) employed a combined dietary and chemical induction approach. The approaches included the use of a high-fat diet plus streptozotocin [31], a high-fat diet plus Olanzapine [59], a high-fat diet plus vitamin D [51], a high-fat diet plus azoxymethane [67], and a high-fat diet plus STZ prepared in citric acid and sodium citrate buffer [56]. This approach leads to the rapid development of MetS features compared to diet-only models because it requires a shorter experimental duration and lower doses of chemicals like STZ, reducing overall costs compared to prolonged diet-only models.

#### 3.4.6. Other Models of Metabolic Syndrome

A study [54] used the approach of utilizing polycystic ovary syndrome (PCOS) rats to model metabolic syndrome. This was achievable because PCOS and MetS are closely interconnected conditions that share overlapping pathophysiological features and risk factors in females of reproductive age. They share features like insulin resistance, hyperinsulinemia, dyslipidemia, obesity, and NAFLD.

### 3.5. Intervention Characteristics

With reference to Table 3, the intervention characteristics are summarized as follows.

#### 3.5.1. Type of Intervention

Among the studies reviewed, a majority (*n* = 28; 59.6%) administered curcumin or its derivatives as a post-treatment intervention, aimed at reversing or mitigating established pathological features [24,26,28,29,32,33,34,35,36,39,43,48,49,53,54,55,56,57,58,59,60,61,62,63,65,66,67,69]. A substantial portion (*n* = 14; 29.8%) employed curcumin as a co-treatment [25,27,30,37,39,44,46,47,48,49,60,64,66,70], administered concurrently with the disease-inducing agent. Pre-treatment administration was reported in three studies [31,41,42], while two studies utilized both pre- and post-treatment [45,50] approaches simultaneously.

Curcumin and its variants (e.g., nano-curcumin), along with extracts, such as turmeric, or combinations with other agents, were used as treatment. It was either administrated alone or in conjunction with other substances (i.e., piperine, metformin, Lovastatin, or probiotics). The majority of the interventions [25,26,29,31,32,34,35,36,37,40,41,42,43,44,45,46,47,48,49,50,51,53,55,56,59,60,65,66,67,68,70] involved pure curcumin (65.95%, *n* = 31), while curcumin extracts, derivatives, or formulations, such as bicurcuma, tetrahydrocurcumin, and nano-curcumin, accounted for 34.05% (*n* = 16) of studies [24,27,28,30,33,38,39,52,54,57,59,61,62,63,64,69]. Common negative controls included HFD- or fructose-fed animals, frequently showing models of MetS without treatment. Some studies employed simple vehicles, such as carboxymethylcellulose (CMC) or saline, to align with treatment protocols [52,69]. Positive controls were missing in most of the studies included in this review. However, controls like Pioglitazone (4 mg/kg or 10 mg/kg) [34,68], Lovastatin [35,50], Atorvastatin [36], or piperine [53] were employed, providing a baseline against which the effects of curcumin could be compared, where available. Also, some studies employed combinations, such as metformin or specific probiotics.

#### 3.5.2. Dosing Strategies

Doses of curcumin and related turmeric formulations administered across the included animal studies varied widely, ranging from 10 µM in cell-equivalent models to 2000 mg/kg in high-dose interventions. The most commonly administered doses were in the range of 50–200 mg/kg, with 100 mg/kg/day appearing in 13 studies (approximately 28%), making it the most frequently used single dose [25,27,40,43,47,49,51,52,54,57,58,64,69]. Standard oral doses of curcumin across studies ranged between 25 and 500 mg/kg, while dietary formulations were expressed in percentage weight/weight (*w*/*w*), such as 0.05%, 0.1%, or 0.7% *w*/*w* [45,46,60,63], and were typically used over longer durations of 10–22 weeks. Some studies also employed topical (transdermal patch) or nano-formulated delivery systems at lower but targeted doses, such as 5 mg/kg for nano-curcumin, or a 4 cm^2^ patch with 200–250 nM diameter curcumin particles [29,58,62].

#### 3.5.3. Treatment Duration

**Figure 5:**
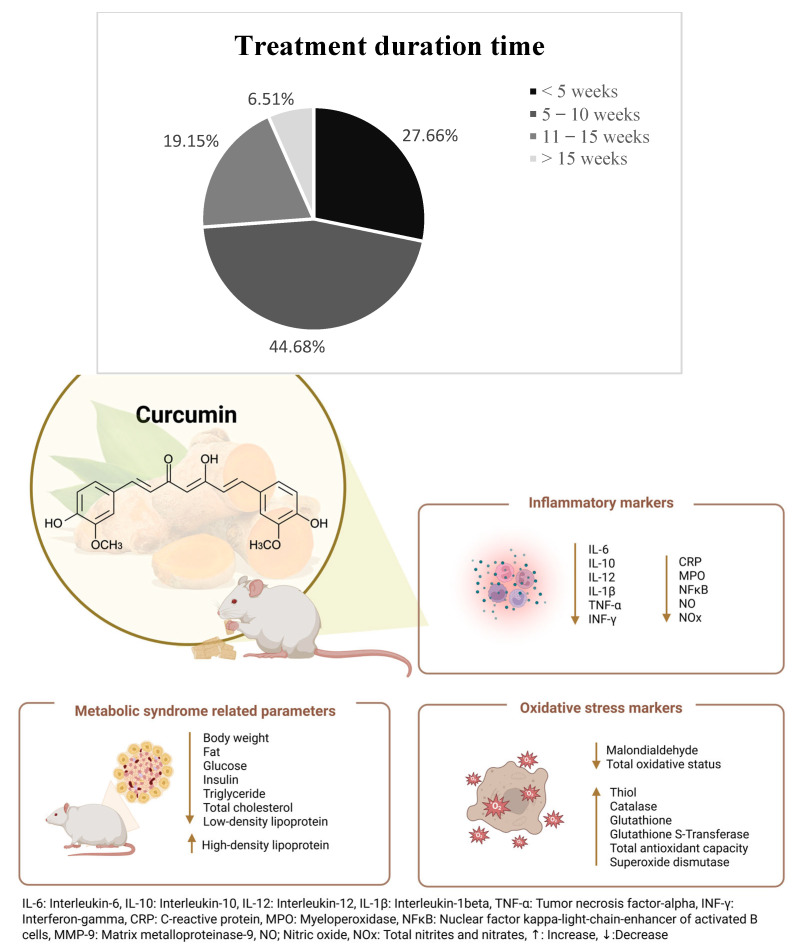
The consumption duration of curcumin/*Curcuma longa* extracts in the rodents (**upper panel**) and their beneficial effects on metabolic-syndrome-related parameters, inflammatory markers, and oxidative stress status (**lower panel**).

Testing durations across the 47 included preclinical studies (as depicted in Figure 5) ranged from 44 h (very short-term pharmacokinetic studies, e.g., [50]) to 24 weeks (long-term exposure studies, e.g., [38]). Most studies, however, favored short to intermediate timelines. An analysis of treatment duration revealed that the majority of studies (53.19%) implemented curcumin or turmeric interventions lasting 4 to 8 weeks [25,28,29,32,33,34,36,39,40,41,42,43,44,50,55,56,58,59,60,61,64,65,66,68,69], consistent with standard protocols for evaluating metabolic outcomes like glucose regulation, lipid profiles, and insulin resistance. A smaller proportion of studies (10.64%) used short-term interventions (<4 weeks) (24, 27, 31, 54, 62], often focusing on acute metabolic or histological responses. Conversely, long-term studies (≥10 weeks) were less common, accounting for 19.15% of included studies [26,30,35,37,38,45,46,47,48,49,50,53,57,63,67,70], and they were typically associated with chronic dietary models or sustained environmental exposures to mimic progressive metabolic syndrome features. Only two studies (4.26%) had testing durations between 5 and 10 weeks that did not fall neatly into the short or long categories [31,66]. This distribution underscores a preclinical research trend toward short to mid-term evaluations in metabolic syndrome models, possibly due to resource and ethical considerations. Longer-duration studies remain crucial for capturing chronic systemic effects, such as liver fibrosis, endothelial dysfunction, and long-term adiposity changes, associated with metabolic syndrome’s progression.

### 3.6. Effects of Curcumin and Curcuma longa Extracts on Metabolic-Syndrome-Related Parameters

Across the 47 studies (Figure 5, Supplementary Tables S4 and S6) included in this systematic review, curcumin and *Curcuma longa* extracts and derivatives consistently demonstrated beneficial effects on a range of metabolic parameters in rodent models of metabolic syndrome. These parameters included body weight gain (WG), adiposity (fat accumulation), blood glucose (Glu), insulin (INS), triglycerides (TG), total cholesterol (TC), low-density lipoprotein cholesterol (LDL), high-density lipoprotein cholesterol (HDL), blood pressure (BP), and markers like hemoglobin A1C (HbA1c) and body mass index (BMI).

#### 3.6.1. Body Weight and Adiposity

Reductions in body weight gain (WG) and fat accumulation were the most frequently reported outcomes (Supplementary Tables S4 and S6). A total of 22 studies reported significant decreases in WG following curcumin or *Curcuma longa* treatment. These include studies by Afifi et al. [25], Ariamoghaddam et al. [29], Auger et al. [30], Ding et al. [34,35], Ejaz et al. [37], Hong et al. [38], Hussein et al. [41], Kelany et al. [44], Kobori et al. [45], Koboziew et al. [46], Lee et al. [47], Li et al. [48], Pan et al. [57], Preez et al. [58], Ramesh et al. [59], Rivego-Sagada et al. [61], Sarker et al. [63], Tiwari-Pandey et al. [66], Wu et al., [67], Zhang et al. [68], and Zou et al. [70]. Similarly, reductions in fat mass were observed in 24.40% (*n* = 11) of studies [25,35,37,45,46,53,55,57,58,66,67]. These outcomes suggest that curcumin may play a role in preventing obesity, a key component of metabolic syndrome.

#### 3.6.2. Glycemic Control (Glucose and Insulin)

Blood glucose levels (Glu) were reduced in 24 studies [25,28,30,34,35,36,37,39,41,43,44,47,50,54,55,56,57,58,59,61,62,64,65,69] in which significant hypoglycemic effects were reported. Likewise, serum insulin levels (INS) were decreased in 16 studies [25,28,34,35,42,43,44,45,47,50,54,55,56,57,61,64], suggesting improved insulin sensitivity.

#### 3.6.3. Lipid Profile (TG, TC, LDL, HDL)

Improvements in serum lipid profiles were reported in the majority of studies (Figure 5, Supplementary Table S7). Specifically, triglyceride (TG) levels were significantly reduced in studies, including those by Abiodun et al. [24], Afifi et al. [25], Amin et al. [28], Ariamoghaddam et al. [29], Auger et al. [30], Bulboacă et al. [31], D’Antongiovanni et al. [32], Ding et al. [34], Ding et al. [35], Eissa et al. [36], Ejaz et al. [37], Hong et al. [38], Hu et al. [39], Hussein et al. [41], Kapar et al. [43], Kelany et al. [44], Kobori et al. [45], Li et al. [48], Li et al. [49], Li et al. [50], Majithiya et al. [52], Neyrinck et al. [55], Pan et al. [57], Pereez et al. [58], Ramesh et al. [59], Rivego-Sagado et al. [61], Sarker et al. [63], Severcan et al. [64], and Zhang et al. [68]. Total cholesterol (TC) reductions were observed in 32 studies, such as those by Abiodun et al. [24], Afifi et al. [25], Amin et al. [28], Ariamoghaddam et al. [29], Auger et al. [30], Bulboacă et al. [31], D’ Antongiovanni et al. [32], Demir [33], Ding et al. [34], Ding et al. [35], Eissa et al. [36], Ejaz et al. [37], Hong et al. [38], Hu et al. [39], Hussein et al. [41], Kapar et al. [43], Kelany et al. [44], Kobori et al. [45], Li et al. [49], Li et al. [50], Li et al. [51], Majithiya et al. [52], Neyrinck et al. [55], Preez et al. [58], Ramesh et al. [59], Rao et al. [60], Rivego-Sagado et al. [51], Sarker et al. [63], Severcan et al. [64], Su et al. [65], Zhang et al. [68], and Zou et al. [70]. Low-density lipoprotein cholesterol (LDL) was decreased in 17 studies [24,25,28,31,35,36,38,39,41,48,51,59,63,64,65,68,70]. High-density lipoprotein cholesterol (HDL) was increased in 20 studies [24,25,28,30,31,35,36,38,41,50,51,59,63,64,65,68,70]. These findings suggest that curcumin may contribute to a favorable lipid balance by both reducing atherogenic lipids and enhancing protective lipoproteins.

#### 3.6.4. Blood Pressure, Cardiovascular Parameters, and Other Metabolic Indicators

Systolic and/or diastolic blood pressure (BP) was reduced in eight studies [31,36,40,44,49,58,64,68]. Given the association of hypertension with metabolic syndrome, these outcomes highlight the potential cardioprotective properties of curcumin. Additional metabolic markers, such as HbA1c, BMI, and non-alcoholic fatty liver disease (NAFLD), were also influenced (Figure 5, Supplementary Tables S4 and S6). D’Antongiovanni et al. [32] reported reductions in HbA1c and BMI, while Lee et al. [47] noted a decrease in NAFLD scores, suggesting broader metabolic benefits beyond glucose and lipid metabolism. Overall, curcumin and *Curcuma longa* extracts showed beneficial modulation of metabolic syndrome parameters in the majority of included studies. These outcomes demonstrate a consistent trend of curcumin-mediated improvement in key markers of metabolic syndrome, supporting its therapeutic potential in preclinical models. However, inter-study variability suggests the need for further research to establish optimal dosing, treatment duration, and bioavailability of curcumin formulations.

### 3.7. Effects of Curcumin and Its Derivatives on Inflammatory Markers in Rodent Models of Metabolic Syndrome

With reference to Figure 5 and Supplementary Table S8, out of the 47 studies included in this systematic review, 18 studies (38.29%) reported data on the modulation of inflammatory markers following administration of curcumin or *Curcuma longa* extracts in rodent models of metabolic syndrome [25,27,28,30,31,32,43,44,45,49,53,54,55,56,64,65,67,68]. These studies evaluated a range of inflammatory cytokines and mediators, such as tumor necrosis factor-alpha (TNF-α), interleukin-6 (IL-6), interleukin-1β (IL-1β), C-reactive protein (CRP), nuclear factor-kappa B (NFκB), interferon-gamma (IFN-γ), myeloperoxidase (MPO), matrix metalloproteinase-9 (MMP-9), and nitric oxide (NO/NOx).

TNF-α was the most frequently assessed pro-inflammatory marker, reported in nine studies. All eight studies [25,30,44,45,49,55,56,64,65] demonstrated a significant reduction in TNF-α levels following curcumin treatment, suggesting a potent anti-inflammatory action. IL-6 was reported in five studies, in which curcumin administration led to decreased IL-6 levels in all studies [25,53,54,55,56,67], reinforcing its suppressive effect on systemic inflammation. Reductions in CRP levels were observed in three studies [25,28,54], supporting the potential of curcumin to alleviate low-grade chronic inflammation typically seen in metabolic syndrome. Three studies [32,53,56] reported significant downregulation of IL-1β in response to curcumin, contributing further evidence of its anti-inflammatory effect at the cytokine level. A significant reduction in IFN-γ levels was noted in two studies [43,45], indicating that curcumin may also suppress Th1-mediated immune responses associated with metabolic dysregulation. Only one study [30] measured NFκB and observed its inhibition, consistent with curcumin’s well-established role as a modulator of transcription factors involved in inflammation.

D’Antongiovanni et al. [32] reported a decrease in MPO activity, indicating reduced neutrophil activation and oxidative inflammatory stress. Li et al. [49] found that MMP-9 expression was significantly downregulated, suggesting a potential role for curcumin in mitigating vascular remodeling and extracellular matrix degradation in metabolic disorders. Reductions in NO or total nitrites/nitrates were reported in three studies [27,31,43]. While NO is also considered an oxidative stress marker, its dual role in inflammation, particularly in endothelial function, justifies its inclusion here. These findings suggest that curcumin and its derivatives exert broad anti-inflammatory effects in rodent models of metabolic syndrome. Most studies reported statistically significant reductions in multiple key inflammatory mediators, particularly TNF-α, IL-6, IL-1β, and CRP, which are central to the pathophysiology of insulin resistance, adipose tissue dysfunction, and cardiovascular complications in metabolic syndrome. The diversity of inflammatory markers and consistent downregulation across studies highlight the multi-targeted anti-inflammatory potential of curcumin, possibly through inhibition of upstream transcription factors, such as NFκB, and modulation of cytokine expression pathways.

### 3.8. Effects of Curcumin and Its Derivatives on Oxidative Stress Markers in Rodent Models of Metabolic Syndrome

Out of the 47 included studies (Figure 5, Supplementary Table S9), 9 studies [25,27,31,33,36,41,44,45,64] specifically investigated the effects of curcumin or *Curcuma longa* extracts on oxidative stress markers in rodent models of metabolic syndrome. These markers are crucial for assessing the redox balance in metabolic syndrome and include malondialdehyde (MDA), superoxide dismutase (SOD), catalase (CAT), glutathione (GSH), total oxidative status (TOS), total antioxidant capacity (TAC), and other antioxidant enzymes.

MDA, a biomarker of lipid peroxidation and cellular damage, was the most frequently assessed oxidative stress parameter. Eight studies [25,27,31,33,36,41,44,45] consistently reported a significant reduction in MDA levels following curcumin treatment, indicating decreased lipid peroxidation and oxidative damage. In terms of antioxidant enzyme activity, curcumin was shown to enhance endogenous antioxidant defenses. Increases in SOD activity were documented in studies by Afifi et al. [25] and Akintunde et al. [27]. Similarly, CAT levels were elevated in two studies [27,33], while GSH and GST, key regulators of cellular redox homeostasis, were upregulated in the studies by Demir et al. [33].

Furthermore, curcumin administration was associated with reduced total oxidative status (TOS) and increased total antioxidant capacity (TAC) in studies by Bulboacă et al. [31] and Severcan et al. [64]. Notably, Bulboacă et al. [31] reported a multi-faceted antioxidant profile, with curcumin increasing TAC and thiol levels while reducing MDA and TOS, suggesting a robust systemic antioxidant response. A minority of studies examined additional oxidative markers, such as NO (nitric oxide) and NOx (total nitrites and nitrates). Akintunde et al. [27] and Kapar et al. [43] reported significant reductions in NO levels, while Bulboacă et al. [31] noted a reduction in NOx, which further supports the role of curcumin in mitigating oxidative nitric stress. While most studies demonstrated curcumin’s antioxidant potential, one study [42] did not report oxidative stress and inflammatory outcomes despite elevated glucose and insulin levels. Overall, the findings from these nine studies indicate that curcumin and its derivatives exert potent antioxidant effects in rodent models of metabolic syndrome. The mechanisms appear to involve both suppression of oxidative damage (e.g., MDA, TOS) and enhancement of endogenous antioxidant defense (e.g., SOD, CAT, GSH, TAC). These effects are consistent with curcumin’s known redox-modulating properties and suggest a beneficial role in mitigating oxidative stress, a key pathological feature of metabolic syndrome.

### 3.9. Meta-Analysis of Curcumin’s Effect on Metabolic Syndrome in Rodent Models

This study presents a detailed synthesis of the quantitative outcomes of studies investigating the therapeutic efficacy of *Curcuma longa* (curcumin) on metabolic-syndrome-related parameters in rodent models. The meta-analysis focused on core physiological markers, including weight gain, glucose and insulin levels, and lipid profiles, namely, total cholesterol (TC), low-density lipoprotein cholesterol (LDL-C), high-density lipoprotein cholesterol (HDL-C), and triglycerides (TG). All analyses compared curcumin-treated groups with appropriate controls, pooling data from studies that met the inclusion criterion of at least three comparable experimental models.

Weight gain was assessed using five rat studies [29,44,58,59,68] and eleven mouse studies [30,37,38,45,46,50,55,57,63,67,70]. The results demonstrated that curcumin statistically caused a significant reduction in weight gain for both rats (MD: −42.10 g, 95% CI: −64.09 to −20.12; *p* < 0.001) and mice (MD: −2.91 g, 95% CI: −4.23 to −1.59; *p* < 0.001). Notably, substantial heterogeneity was observed (rats: I^2^ = 98.1%, mice: I^2^ = 95%), indicating inter-study variability, likely due to differences in dose, duration, or animal strain.

For glucose levels, ten rat studies, e.g., [39,44], and nine mouse studies, e.g., [57,62,69], were included. Curcumin significantly reduced glucose levels in both rats (MD: −55.59 mg/dL, 95% CI: −91.40 to −19.77; *p* < 0.001) and mice (MD: −28.69 mg/dL, 95% CI: −51.86 to −5.51; *p* < 0.001). Despite the significant effect, both models showed high heterogeneity (I^2^ = 100%). Egger’s test revealed no significant publication bias. The analysis of insulin levels involved three rat studies and six mouse studies. While reductions in insulin levels were observed (rats: MD: −10.74 mg/dL, mice: MD: −11.16 mg/dL; *p* < 0.001), these did not reach statistical significance (*p* > 0.05). High heterogeneity (I^2^ = 100%) was present in both groups. Funnel plots and Egger’s test showed no evidence of publication bias.

A total of 21 studies (12 in rats, 9 in mice) reported curcumin effects on TC. Both species showed significant reductions in rats (MD: −35.77 mg/dL, 95% CI: −45.79 to −25.75; *p* < 0.001) and mice (MD: −52.61 mg/dL, 95% CI: −73.51 to −31.70; *p* < 0.001). Heterogeneity was high (I^2^ = 98%). Egger’s test suggested potential publication bias in mouse models (*p* = 0.025), but not in rats. For LDL-C, curcumin significantly lowered levels in rats (MD: −69.34 mg/dL, 95% CI: −95.32 to −43.35; *p* < 0.001) and mice (MD: −42.93 mg/dL, 95% CI: −51.08 to −34.77; *p* < 0.001). Heterogeneity was substantial in rats (I^2^ = 100%) but negligible in mice. No publication bias was observed based on funnel plots and Egger’s test. Curcumin significantly increased HDL-C in rats (MD: 11.57 mg/dL, 95% CI: 6.84 to 16.31; *p* < 0.001), but not in mice (MD: −853.1 mg/dL, 95% CI: −3581.93 to 1875.73; *p* < 0.001). While rat studies showed meaningful improvement, both models exhibited high heterogeneity. No significant publication bias was evident. Curcumin treatment resulted in reduced TG levels in both rats (MD: −70.17 mg/dL, 95% CI: −96.62 to −43.73; *p* < 0.001) and mice (MD: −24.57 mg/dL, 95% CI: −35.36 to −13.77; *p* < 0.001), with both achieving statistical significance. High heterogeneity (rats and mice: I^2^ > 95%) was noted.

Funnel plots for all outcomes generally showed moderate to symmetrical distribution, indicating limited publication bias. Slight asymmetries observed in weight gain and TC outcomes suggest minor small-study effects or methodological variation. These observations are consistent with the existing literature, including Hooijmans et al. [23] and Sena et al. [72], who report methodological diversity as a primary source of heterogeneity in preclinical meta-analyses. The meta-analysis indicates curcumin’s beneficial effects on multiple metabolic syndrome parameters, particularly weight gain, glucose, total cholesterol, LDL-C, and TG levels. Although insulin and HDL-C outcomes were more variable, the overall findings support curcumin as a promising candidate for managing metabolic syndrome in preclinical settings.

## 4. Discussion

The comprehensive systematic review and meta-analysis offer compelling evidence supporting the therapeutic efficacy of *Curcuma longa* Linn. (curcumin) in ameliorating a broad array of metabolic syndrome (MetS)-related parameters in rodent models. The consistent modulation of body weight, glycemic indices, lipid profiles, and blood pressure across studies underscores curcumin’s potential not only as a pharmacological agent but also as a functional food compound for the prevention and management of MetS. These outcomes consolidate and extend prior preclinical findings that attribute curcumin’s broad-spectrum metabolic benefits to its anti-inflammatory, antioxidant, and insulin-sensitizing actions [67,68].

*Curcuma longa*, mainly through its active compound curcumin, has been reported to exert diverse biological effects relevant to metabolic syndrome. A key mechanism is its anti-inflammatory action via inhibition of the NF-κB pathway, which reduces pro-inflammatory cytokines, such as TNF-α, IL-6, and IL-1β. Rodent studies have shown that curcumin suppresses NF-κB activation in adipose and hepatic tissues, thereby mitigating chronic inflammation associated with MetS [73,74]. Curcumin also enhances antioxidant defenses by activating the Nrf2 pathway, leading to increased expression of enzymes like SOD, catalase, and glutathione peroxidase that are essential in countering oxidative stress and insulin resistance [75]. It improves insulin sensitivity through IRS-1 phosphorylation and PI3K/Akt pathway activation, promoting glucose uptake and reducing hepatic gluconeogenesis [76,77]. In lipid metabolism, curcumin suppresses lipogenesis via downregulation of SREBP-1c and FAS and upregulates PPAR-α, enhancing β-oxidation [37,78]. Curcumin’s anti-obesity effects arise from its ability to inhibit adipogenesis and lipid buildup, mainly by downregulating adipogenic transcription factors, such as PPARγ and C/EBPα [37]. Moreover, curcumin encourages fatty acid oxidation and blocks the activity of lipogenic enzymes like acetyl-CoA carboxylase, which helps reduce lipid storage. These molecular actions lead to decreased body weight and fat, which are important for reducing central obesity, a key factor in insulin resistance and systemic inflammation in MetS. It also favorably modulates lipid profiles by lowering serum total cholesterol, LDL, and triglycerides while increasing HDL levels. Mechanistically, this is achieved through the suppression of HMG-CoA reductase and the enhanced expression of LDL receptors [69], as well as the inhibition of intestinal lipid absorption and the stimulation of reverse cholesterol transport [70]. These changes improve lipid homeostasis and reduce cardiovascular risk.

Improvements in glycemic regulation are consistently observed and mechanistically linked to curcumin’s ability to enhance insulin receptor phosphorylation, facilitate GLUT4 translocation, and preserve pancreatic β-cell integrity [70]. Moreover, by mitigating oxidative stress and inhibiting the NF-κB pathway, curcumin enhances insulin sensitivity and lowers fasting glucose and HbA1c levels [75,79]. These effects are robust across models, although bioavailability challenges and dosing variability account for some inter-study inconsistencies. Although studied less frequently, curcumin’s capacity to lower systolic and diastolic blood pressure reflects its vasoprotective effects. These are mediated by upregulation of endothelial nitric oxide synthase (eNOS), improved nitric oxide bioavailability, and reduced vascular oxidative stress [80,81]. Enhanced arterial compliance resulting from these mechanisms offers cardioprotective benefits, particularly pertinent in MetS-related hypertension.

Chronic low-grade inflammation, marked by elevated cytokines, such as TNF-α, IL-6, and CRP, is a key feature of MetS. Curcumin’s anti-inflammatory efficacy is underpinned by its inhibition of the NF-κB signaling axis, which governs the transcription of pro-inflammatory mediators [73,82]. By preventing NF-κB activation and nuclear translocation, curcumin significantly suppresses cytokine production, thereby attenuating adipose tissue inflammation and improving metabolic outcomes. In addition, curcumin downregulates other inflammatory mediators, including MPO and MMP-9, while modulating nitric oxide metabolites, thereby targeting both inflammatory and oxidative pathways involved in endothelial dysfunction [83]. This systemic anti-inflammatory action is especially advantageous in a disorder like MetS, where immune dysregulation underlies multiple clinical features.

The antioxidant properties of curcumin are supported by consistent reductions in malondialdehyde (MDA) and total oxidative status (TOS), alongside elevated levels of endogenous antioxidants, such as SOD, CAT, and GSH. Mechanistically, curcumin activates the Nrf2 transcription factor, which upregulates phase II detoxifying enzymes and antioxidant proteins [84,85]. This redox-modulatory capacity helps restore oxidative balance, reduce lipid peroxidation, and protect against β-cell and endothelial damage.

Furthermore, curcumin decreases levels of nitric oxide and its reactive derivatives (NOx), likely through the suppression of iNOS and the inhibition of peroxynitrite formation [86]. These actions mitigate nitrosative stress, a critical yet underappreciated contributor to vascular complications in MetS, and suggest broader protection against MetS-associated comorbidities, such as NAFLD, atherosclerosis, and type 2 diabetes [87]. Despite compelling preclinical data, curcumin’s translation to human therapy is impeded by its poor oral bioavailability. Factors like rapid metabolism and low aqueous solubility limit systemic exposure. However, innovative delivery systems, including nanoparticles, liposomes, and co-administration with piperine, have demonstrated enhanced bioavailability and hold promise for clinical application [88,89].

Furthermore, these findings are promising, particularly in the context of curcumin being a functional food, where multi-target actions and safety profiles are critical for long-term dietary interventions. Curcumin’s broad range of health-promoting effects supports its classification as a functional food, rather than solely a pharmacological agent [38,39,41,44,45,46,59,63]. These outcomes suggest that turmeric plays a preventive and regulatory role in maintaining overall physiological balance, rather than merely addressing specific disease symptoms as pharmacologic agents do. Its effectiveness across multiple biological pathways, including lipid and glucose metabolism, inflammation suppression, and oxidative stress reduction, reflects the multi-targeted mechanisms typical of functional foods. Furthermore, the consistency of these beneficial effects across a wide range of studies and experimental conditions highlights the reliability of turmeric’s health impact. Additionally, as a widely consumed culinary spice with a well-established safety profile, turmeric can be easily incorporated into daily diets, making it a practical option for long-term health maintenance and disease prevention. Unlike pharmacological agents, which are often limited to short-term use and prescribed under clinical supervision, functional foods like *Curcuma longa* offer a sustainable approach to supporting metabolic health and reducing the risk of chronic disease. Therefore, the collective evidence positions curcumin not just as a therapeutic compound but as a valuable dietary component with significant potential in preventive nutrition and functional food applications [53,65,66].

The predominance of post-treatment administration of curcumin in the reviewed studies highlights a strong research focus on its therapeutic potential, particularly its capacity to alleviate established features of metabolic syndrome (MetS), such as insulin resistance, dyslipidemia, hypertension, and inflammation. This reflects real-world clinical settings, where interventions typically begin after the onset of disease. A notable number of studies also employed curcumin as a co-treatment, suggesting interest in its role as a protective adjunct capable of moderating disease progression during ongoing metabolic stress. Although less common, pre-treatment studies are crucial in assessing curcumin’s preventive value, as they explore its ability to enhance cellular defense and reduce susceptibility to MetS in high-risk individuals. Additionally, a few studies utilized both pre- and post-treatment, offering insights into curcumin’s broad protective window across different stages of disease development. The diverse timing of curcumin administration underscores its versatile potential in both the prevention and management of metabolic syndrome, supporting its relevance in therapeutic and public health strategies. However, of note in the studies included is the relatively short duration of most interventions, which may not fully capture the long-term effects of *Curcuma longa* on the progression and management of metabolic syndrome. While short-term studies can provide important preliminary insights, metabolic syndrome is a chronic condition that develops and evolves over time. Therefore, long-duration interventions are essential to evaluate both the sustained efficacy and the potential cumulative or delayed effects, whether therapeutic or adverse, of *Curcuma longa* as a functional food. Future preclinical studies should prioritize extended intervention periods to better mimic the chronic nature of the syndrome and to provide more translationally relevant data for potential clinical applications.

Environmental variables like housing temperature, light–dark cycles, humidity, and feeding schedules are critical in shaping metabolic phenotypes in rodent models, yet they remain underreported. In our review, fewer than half of the included studies detailed housing temperature or feeding conditions, and only a third reported humidity levels. While a 12:12 light–dark cycle and ad libitum feeding were most commonly mentioned when noted, inconsistent reporting introduces confounding factors. Evidence shows that even modest shifts in environment can alter outcomes. For instance, sub-thermoneutral housing can activate brown adipose tissue and affect glucose metabolism, potentially exaggerating effects attributed to *Curcuma longa* [90,91]. Disruptions in circadian rhythms via irregular light cycles or feeding times also impact insulin sensitivity, lipid metabolism, and inflammation [92,93]. Given *Curcuma longa*’s proposed roles in reducing inflammation, improving lipid profiles, and enhancing insulin sensitivity, failure to account for or report these variables raises concerns about internal validity. Variations in energy expenditure or glucose regulation due to environmental conditions may confound observed benefits. These gaps emphasize the importance of following reporting standards to ensure reproducibility and clarify the true therapeutic potential of *Curcuma longa.*

The findings from this meta-analysis provide growing preclinical support for the metabolic health benefits of *Curcuma longa* (turmeric), particularly in its capacity to modulate parameters associated with metabolic syndrome. The results underscore curcumin’s potential to reduce key biomarkers, such as body weight, glucose, insulin, cholesterol, and triglyceride levels, in rodent models. Importantly, these findings align with existing literature that positions curcumin as a promising nutraceutical with pleiotropic metabolic effects. While the results appear generally favorable, their interpretation must consider several methodological and translational limitations. One central issue is the heterogeneity observed across almost all analyzed outcomes. This variation likely arises from differences in curcumin formulation (e.g., raw turmeric vs. nanoparticle-enhanced forms), dose, duration of administration, and animal model characteristics. Previous reviews of preclinical nutrition studies have also reported that experimental design variability contributes significantly to statistical heterogeneity [23]. Therefore, while curcumin consistently trends toward beneficial effects, the magnitude and consistency of its efficacy may differ substantially based on how, when, and in what form it is administered.

Methodological heterogeneity across studies, such as the wide range of *Curcuma longa* dosages reported in preclinical rodent studies (from 10 µM to 2000 mg/kg), reflects variations in formulation, administration routes, and treatment durations. While converting these doses to human equivalent doses (HEDs) could aid clinical translation, such scaling involves assumptions that may not hold consistently across diverse study designs. Maintaining the original animal dosages preserves the integrity of the preclinical data and allows for direct comparison within the animal model context. Nonetheless, understanding this dosage variability is crucial for interpreting potential human applications, and future research should aim to bridge these preclinical findings with well-designed clinical trials to establish safe and effective dosing regimens in humans.

In examining publication bias, funnel plot analyses generally indicated moderate to good symmetry, suggesting a low risk of systematic bias across most included outcomes. However, subtle funnel asymmetry observed in parameters like triglycerides, LDL-C, and weight gain may point to small-study effects or reporting bias, which are common challenges in animal research [66]. These deviations can reflect either overestimation of treatment effects in underpowered studies or methodological inconsistencies that skew reported outcomes. Nonetheless, the absence of egregious bias strengthens the reliability of the pooled estimates, albeit with caution.

The notable inconsistency in findings across insulin and glucose parameters further highlights the complexity of curcumin’s pharmacodynamics. Although many studies demonstrate hypoglycemic or insulin-sensitizing effects, a few report negligible or even contradictory outcomes. This may relate to curcumin’s limited bioavailability, which has been documented to affect systemic absorption and tissue distribution [80]. Formulations with enhanced bioavailability (e.g., liposomal or piperine-combined preparations) may be more effective, but not all studies in this meta-analysis standardized such preparations, potentially diluting the observed effect sizes.

Although none of the rodent studies included in this review reported adverse effects associated with turmeric or curcumin administration, it is important to contextualize these findings within the broader safety landscape. Clinical studies have generally found turmeric and curcumin to be well tolerated, even at relatively high doses. However, there have been isolated reports of hepatotoxicity in humans, some linked to curcumin supplements [94,95,96]. Consequently, caution is advised in individuals with pre-existing hepatobiliary dysfunction or gallstones, as well as those taking hepatically metabolized medications. These considerations underscore the importance of rigorous safety evaluation before translating preclinical findings into human recommendations.

The results of the current study are promising, with a few limitations. A major area of emphasis highlighted by our review is the considerable variability in the experimental designs across included studies. Rodent models of metabolic syndrome were induced using a range of protocols, including high-fat diets, high-fructose diets, streptozotocin administration, or combinations thereof, with differing durations and diagnostic criteria. This heterogeneity complicates direct comparisons and may partially account for variations in the reported outcomes. To enhance reproducibility and the interpretability of findings, there is a pressing need for greater standardization in preclinical research. Harmonizing induction protocols, establishing consistent diagnostic criteria for metabolic syndrome in rodents, and adopting unified outcome measures would enable more robust cross-study comparisons and strengthen the translational relevance of preclinical evidence, and future studies should consider adopting consensus guidelines or frameworks to reduce methodological variability. Also, the included data were derived exclusively from preclinical rodent models, which may limit the generalizability of the findings to human populations due to physiological differences. Additionally, despite the funnel plot analysis showing minimal publication bias, the potential for small-study effects and underreporting of negative results cannot be fully ruled out. Lastly, the absence of standardized reporting across studies limited the ability to conduct subgroup analyses or assess dose–response relationships, which are critical for translational relevance.

## 5. Conclusions

The systematic review and meta-analysis provide strong preclinical evidence supporting the therapeutic potential of *Curcuma longa* as a functional food ingredient for the prevention and management of metabolic syndrome. Curcumin, its primary bioactive compound, exerts pleiotropic effects on key metabolic parameters, including body weight, lipid profiles, glucose metabolism, oxidative stress, and inflammation. These actions were consistent and statistically significant across studies, with minimal evidence of publication bias. Although moderate heterogeneity was observed, the overall findings reinforce curcumin’s promise as a natural, multi-target intervention for metabolic disorders. Importantly, curcumin’s anti-inflammatory, antioxidant, and insulin-sensitizing properties align well with the pathophysiological mechanisms of metabolic syndrome. Its incorporation into the diet through functional foods, such as turmeric-enriched meals, curcumin-fortified beverages, or encapsulated supplements, offers a practical route for population-level health benefits, as the studies show that regular intake of curcumin-enriched products can beneficially modulate glucose levels, lipid metabolism, and oxidative biomarkers. Future functional food development should focus on improving bioavailability through optimized formulations, including nanoparticles or phospholipid complexes. However, addressing challenges related to formulation, standardization, and translational research remains critical for advancing curcumin from bench to bedside.

## Figures and Tables

**Table 1:** Eligibility criteria for the selected studies.

Inclusion Criteria	
Patient/Population	Rodent models of metabolic syndrome.
Intervention	Curcumin, *Curcuma longa*, diferuloylmethane without any combination of other drugs or chemicals, and other types of intervention.
Comparison/Control	Both the effectiveness of curcumin or isolated compounds compared with placebo and/or control.
Outcomes	Metabolic-syndrome-related parameters: lipid profile, glucose level, blood pressure, cardiovascular diseases, type 2 diabetes, insulin resistance, NAFLD. Inflammation markers: interleukin IL-6, IL-1b, tumor necrosis factor (TNF-alpha), etc. Oxidative stress markers: MDA, ROS, GSH levels, etc.
**Exclusion Criteria**	
Studies conducted in vitro Non-curcumin clinical research Non-English, non-experimental Reviews, commentaries, and unpublished studies Publications without full text access and studies lacking relevant outcomes	

**Table 2:** General characteristics of the included studies.

Author	Year	Country	No. of Animals (Total)	Per Group	Rodent Type	Sex	Age (Weeks)
Abiodun et al. [24]	2023	Nigeria	30	5	Wistar	Male	NM
Afifi et al. [25]	2014	Egypt	NM	14–16	Albino Wistar	Male	NM
Ahmed et al. [26]	2020	Saudi Arabia	NM	7–8	Wistar	Male	6–8
Akintunde et al. [27]	2019	Nigeria	40	8	Wistar	Male	7
Amin et al. [28]	2015	Pakistan	NM	6–8	Sprague Dawley	Male/ Female	NM
Ariamoghaddam et al. [29]	2018	Iran	18	6	Rat	NM	8
Auger et al. [30]	2018	France	48	12	C57BL/6	Female	7
Bulboacă et al. [31]	2016	Romania	50	10	Wistar	Male	NM
D’Antongiovanni et al. [32]	2023	Italy	100	10	C57BL/6	Male	5
Demir [33]	2021	Turkey	63	7	Wistar Albino	NM	8–10
Ding et al. [34]	2015	China	NM	6–8	Sprague Dawley	Male	8–10
Ding et al. [35]	2016	China	40	8	C57BL/6	Male	6
Eissa et al. [36]	2021	Egypt	50	10	Sprague Dawley	Male	8
Ejaz et al. [37]	2009	USA	18	6	C57BL/6	Male	4
Hong et al. [38]	2023	China	24	8	CD-1	Male	5
Hu et al. [39]	2013	China	30	10	Sprague Dawley	Male	12.9
Hu et al. [40]	2017	China	42	6	SHRs	Male	8–10
Hussein et al. [41]	2024	Egypt	60	7 and 10	Sprague Dawley	Male	NM
Ibrahim et al. [42]	2019	South Africa	128	31–32	Sprague Dawley	Male/ Female	0.9
Kapar et al. [43]	2020	Turkey	50	10	Sprague Dawley	Male	10–12
Kelany et al. [44]	2016	Egypt	30	10	Sprague Dawley	Male	NM
Kobori et al. [45]	2018	Japan	27	9	C57BL/6J	Male	5
Koboziev et al. [46]	2020	USA	20	10	C57BL/6	Male	5
Lee et al. [47]	2020	South Korea	60	10	C57BL/6	Male	8
Li et al. [48]	2021	China	30	6	C57BL/6 Slac	Male	4
Li et al. [49]	2021	China	45	15	Wistar	Male	9
Li et al. [50]	2015	China	30	10	C57BL/6	Male	6
Li et al. [51]	2015	China	32	8	SHRs, WKY	Male	8–10
Majithiya et al. [52]	2004	India	36	6	Swiss Albino	Male	NM
Miyazawa et al. [53]	2018	USA	47	9–10	C57BL/6	Male	8
Mohammadi et al. [54]	2017	Iran	90	18	Wistar	Female	NM
Neyrinck et al. [55]	2021	Belgium	36	9	B6.V-Lep ob/ob JRj	Male	6
Omaima and Fouad [56]	2009	Egypt	50	10	Albino rat	Male	NM
Pan et al. [57]	2018	China	40	8	C57BL/6J	Male	4
Preez et al. [58]	2019	Australia	120	12	Wistar	Male	8–9
Ramesh et al. [59]	2012	India	60	6	Sprague Dawley	Male	NM
Rao et al. [60]	1970	India	NM	NM	Albino Wistar	Female	6.4
Rivero-Salgado et al. [61]	2024	Mexico	NM	6–8	Wistar	Male	3
Samadder et al. [62]	2024	India	30	6	Swiss Albino	NM	6–8
Sarker et al. [63]	2019	Bangladesh	40	8	Swiss Albino	NM	7.5
Severcan et al. [64]	2021	Turkey	24	6	Wistar Albino	Male	NM
Su et al. [65]	2017	China	60	15	Sprague Dawley	Male	NM
Tiwari-Pandey et al. [66]	2009	Canada	NM	12–15	129T2/SV EmsJ Fshr+/−, WT	Male/ Female	NM
Wu et al. [67]	2021	USA	110	21	FVB	Male	4
Zhang et al. [68]	2012	China	50	7	Sprague Dawley	Male	NM
Zhong et al. [69]	2022	China	16	8	C57BL/6J	Male	8
Zou et al. [70]	2018	China	20	10	ApoE−/−	Male	8

**Table 3:** Experimental designs and treatment protocols for animal models of metabolic syndrome used in the eligible studies.

Author, Year	Type of Treatment	Dose(s) of Treatment	Testing Duration (Weeks)
Abiodun et al. [24]	*Curcuma longa* ethanol extract	1.5, 2, 2.5 g/kg	2
Afifi et al. [25]	Curcumin	40, 80, 100, 200 mg/kg	8
Ahmed et al. [26]	Curcumin	10, 30 µM	12
Akintunde et al. [27]	Curcumin dissolved in olive oil	50, 100 mg/kg	2
Amin et al. [28]	Turmeric	1.5, 3 g/kg	6
Ariamoghaddam et al. [29]	Curcumin (transdermal patch)	4 cm^2^ patch loaded with 200–250 nM	6
Auger et al. [30]	Diet containing 0.05% (*w*/*w*) Biocurcuma™ (curcumin)	NM	22
Bulboacă et al. [31]	Curcumin	1 g/kg	2 (+3 days)
D’ Antongiovanni et al. [32]	Curcumin	49 mg/kg/day	4
Demir [33]	Curcumin dissolved in olive oil	1 mg/kg bw	8
Ding et al. [34]	Curcumin	15, 30, 60 mg/kg	6
Ding et al. [35]	Curcumin	40 mg/kg/day	12
Eissa et al. [36]	Curcumin	200 mg/kg/day	8
Ejaz et al. [37]	Curcumin	500 mg/kg	12
Hong et al. [38]	0.5 mg/kg Bisphenol A + 0.1% (*w*/*w*) curcumin	1000 mg/kg	24
Hu et al. [39]	Curcumin suspended in 0.1% cellulose	200 mg/kg/day	8
Hu et al. [40]	Curcumin (200 µL)	25, 50, 100, 200, 400 mg/kg	8 (every 2 days)
Hussein et al. [41]	Curcumin	80 mg/kg	8
Ibrahim et al. [42]	Curcumin	500 mg/kg bm	6
Kapar et al. [43]	Curcumin	100 mg/kg/day	4
Kelany et al. [44]	Curcumin	200 mg/kg/day	8
Kobori et al. [45]	Curcumin	0.1% *w*/*w*	14
Koboziew et al. [46]	Curcumin powder	0.7% *w*/*w*	13
Lee et al. [47]	Curcumin	100 mg/kg/day	13
Li et al. [48]	Curcumin	2000 mg/kg	10
Li et al. [49]	Curcumin	100, 300, 400 mg/kg	12 (once every 2 days)
Li et al. [50]	Curcumin	40, 80 mg/kg	12
Li et al. [51]	Curcumin	100 mg/kg/day	4
Majithiya et al. [52]	Curcumin in 0.5% sodium carboxymethyl cellulose suspension	100, 200, 400 mg/kg	44 h
Miyazawa et al. [53]	Curcumin	1 g/kg	10 (phase 2) and 20 (phase 3)
Mohammadi et al. [54]	Curcumin prepared at 100 mmol/L in DMSO	100, 300 mg/kg	2
Neyrinck et al. [55]	Curcumin	0.3% curcumin	4
Omaima and Fouad [56]	Curcumin	200 mg/kg	6
Pan et al. [57]	Tetrahydrocurcumin	20, 100 mg/kg	10
Preez et al. [58]	Curcumin suspension	5, 100 mg/kg/day	8
	Curcumin nanoparticles	5 mg/kg/day	8
Ramesh et al. [59]	Curcumin	50 mg/kg	4
Rao et al. [60]	Curcumin	0.10%, 0.25%, 0.50%	7
Rivego-Sagado et al. [61]	Hypercaloric diet with functional food containing turmeric solution	NM	8
Samadder et al. [62]	Curcumin	50 mg/kg	1
	Nano-curcumin-1	25 mg/kg	1
	Nano-curcumin-2	12.5 mg/kg	1
Sarker et al. [63]	Curcumin in drinking water	1%, 2%, 3% *w*/*v*	10
Severcan et al. [64]	Curcumin dissolved in olive oil	100, 200 mg/kg	8
Su et al. [65]	Curcumin	250 mg/kg	8
Tiwari-Pandey et al. [66]	Curcumin	25 mg/kg/day	5 (+7 days)
Wu et al. [67]	Curcumin	2000 mg/kg	15
Zhang et al. [68]	Curcumin	15, 30, 60 mg/kg	4
Zhong et al. [69]	Curcumin in 0.5% carboxymethylcellulose	100 mg/kg/day	4
Zou et al. [70]	Curcumin	1000 mg/kg	16

## Data Availability

All relevant data are within the paper.

## Supplementary Materials

The following supporting information can be downloaded at https://www.mdpi.com/article/10.3390/biomedicines13081911/s1, Supplementary Table S1: Database search strategy for in vivo experiments investigating the beneficial effects of curcumin/Curcuma longa extract consumption on metabolic syndrome. Supplementary Table S2: Assessment of methodological quality of the studies included using the 10-item CAMARADES checklist. Table S3: Risk of bias assessment of the included studies using the SYRCLE tool. Supplementary Table S4: Strategies for inducing metabolic syndrome in animal models across the included studies. Supplementary Table S5: Environmental/feeding conditions of animal models included in the studies. Supplementary Table S6: The summary of outcomes related to parameters associated with metabolic syndrome, inflammation, and oxidative stress highlights the effects of curcumin/*Curcuma longa* extracts in rodent models. Supplementary Table S7: Evaluating the effects of curcumin/*Curcuma longa* extract consumption on weight gain (WG), BMI, insulin (INS), glucose (Glu), HbA1c, and blood pressure (BP) in rodent models of metabolic syndrome. Supplementary Table S8: Evaluating the effects of curcumin/Curcuma longa extract consumption on fat content, lipid profiles, and NAFLD score in rodent models of metabolic syndrome. Supplementary Table S9: Assessing outcomes related to inflammatory markers, including interleukin-6 (IL-6), interleukin-10 (IL-10), interleukin-12 (IL-12), interleukin-1beta (IL-1β), tumor necrosis factor-alpha (TNF-α:), and interferon-gamma (INF-γ), highlights the effects of kefir and its active components in rodent models of metabolic syndrome. Supplementary Table S10: Assessing outcomes related to oxidative stress markers highlights the effects of curcumin/Curcuma longa extract consumption in rodent models of metabolic syndrome. Supplementary Figure S1: Forest plot analysis showing the effects of consuming curcumin/*Curcuma longa* extracts compared to the control group on weight gain (A), glucose level (B), and insulin level (C) in rat (left panel) and mouse (right panel) models. Supplementary Figure S2: A funnel plot illustrating the distribution of publication biases associated with the consumption of curcumin/*Curcuma longa* extracts on weight gain (A), glucose levels (B), and insulin levels (C) in rat (left panel) and mouse (right panel) models. Supplementary Figure S3: A forest plot illustrating the distribution of publication biases associated with the consumption of curcumin/*Curcuma longa* extracts on total cholesterol (A), low-density lipoprotein cholesterol (B), high-density lipoprotein cholesterol (C), and triglyceride (D) levels in the rat (left panel) and mouse (right panel) models. Supplementary Figure S4: A funnel plot illustrating the distribution of publication biases associated with the consumption of curcumin/*Curcuma longa* extracts on total cholesterol (A), low-density lipoprotein cholesterol (B), high-density lipoprotein cholesterol (C), and triglyceride (D) levels in the rat (left panel) and mouse (right panel) models.

## Abbreviations

The following abbreviations are used in this manuscript:

BMI	Body mass index
BP	Blood pressure
CAT	Catalase
CCNP	Curcumin nanoparticle
CRP	C-reactive protein
GLU	Glucose
GSH	Glutathione
GST	Glutathione S-Transferase
Hb1Ac	Hemoglobin A1C
HDL	High-density lipoprotein
HFD	High-fat diet
IL-10	Interleukin-10
IL-12	Interleukin-12
IL-1β	Interleukin-1beta
IL-6	Interleukin-6
INF-γ	Interferon-gamma
INS	Insulin
LDL	Low-density lipoprotein
MDA	Malondialdehyde
MMP-9	Matrix metalloproteinase-9
MPO	Myeloperoxidase
NAFLD	Non-alcoholic fatty liver disease
NCUR	Nano-curcumin
NFκB	Nuclear factor kappa-light-chain-enhancer of activated B cells
NO	Nitric oxide
NOx	Total nitrites and nitrates
SOD	Superoxide dismutase
TAC	Total antioxidant capacity
TC	Total cholesterol
TG	Triglyceride
TNF-α	Tumor necrosis factor-alpha
TOS	Total oxidative status
WG	Weight gain
