# Supplementary material for: Preclinical Evidence of Curcuma longa Linn. as a Functional Food in the Management of Metabolic Syndrome: A Systematic Review and Meta-Analysis of Rodent Studies
Source: Biomedicines. 2025 Aug 5;13(8):1911. doi: 10.3390/biomedicines13081911 (PMC12384037; doi:10.3390/biomedicines13081911)
Supplement: Supplementary file 1 [file biomedicines-13-01911-s001.zip › biomedicines-3785894-supplementary.pdf]

Supplementary Table S1: Database search strategy for *in vivo* experiments investigating the beneficial effects of curcumin/*Curcuma longa extracts* consumption on metabolic syndrome.

Database	Keywords	Results
PubMed<br><br>04/10/2024	KP-1: ((curcumin[Title]) OR (*Curcuma longa* [Title]) OR (diferuloylmethane[Title])) AND ("in vivo"[Title/Abstract]) OR (rat[Title/Abstract])) OR (mice[Title/Abstract]) AND ((metabolic syndromes[Title/Abstract]) AND (insulin resistance[Title/Abstract]) AND (type 2 diabetes[Title/Abstract])	13
	KP-2: (curcumin[Title/Abstract]) OR (*Curcuma longa* [Title/Abstract]) OR (diferuloylmethane[Title/Abstract])) AND ("in vivo"[Title/Abstract]) OR (rat[Title/Abstract])) OR (mice[Title/Abstract]) AND ((metabolic syndromes[Title/Abstract]) AND (obesity[Title/Abstract]) OR (abdominal obesity[Title/Abstract]) AND (hyperlipidemia[Title/Abstract])	64
	KP-3: (((curcumin[Title/Abstract] OR curcum[Title/Abstract]) OR (diferuloylmethane[Title/Abstract])) AND (rat[Title/Abstract]) OR (mice[Title/Abstract]) OR ("in vivo"[Title/Abstract])) AND ((metabolic syndromes[Title/Abstract]) AND (hypertension[Title/Abstract] OR (high blood pressure [Title/Abstract] AND (cardiovascular diseases[Title/Abstract])	511
	KP-4: ((curcumin[Title/Abstract]) OR (curcum longa[Title/Abstract]) OR (diferuloylmethane[Title/Abstract])) AND ("in vivo"[Title/Abstract]) OR (rat [Title/Abstract])) OR (mice[Title/Abstract]) AND ((metabolic syndromes [Title/Abstract]) OR (heart diseases [Title/Abstract]) AND (hypertension [Title/Abstract]) AND (stroke[Title/Abstract])	141
	KP-5: ((curcumin[Title/Abstract]) OR (curcum[Title/Abstract]) OR (diferuloylmethane[Title/Abstract])) AND ("in vivo"[Title/Abstract]) OR (rat [Title/Abstract])) OR (mice[Title/Abstract]) AND ((metabolic syndromes[Title/Abstract]) AND (Abnormal cholesterol levels[Title/Abstract]) OR (cholesterol profile[Title/Abstract]) AND (hypercholesterolemia[Title/Abstract])	5

Database	Keywords	Results
Scopus 02/10/2024	KS-1: (TITLE-ABS-KEY (curcumin) OR TITLE-ABS-KEY (diferuloylmethane) OR TITLE-ABS-KEY AND TITLE-ABS-KEY ("in vivo") OR TITLE-ABS-KEY (rat) OR TITLE-ABS-KEY (mice) AND TITLE-ABS-KEY (metabolic syndromes) AND TITLE-ABS-KEY (insulin resistance) OR TITLE-ABS-KEY (type 2 diabetes) OR TITLE-ABS-KEY (hyperglycemia)	19
	KS -2 : (TITLE-ABS-KEY (curcumin) OR TITLE-ABS-KEY (diferuloylmethane) OR TITLE-ABS-KEY AND TITLE-ABS-KEY ("in vivo") OR TITLE-ABS-KEY (rat) OR TITLE-ABS-KEY (mice) AND TITLE-ABS-KEY (metabolic syndromes) AND TITLE-ABS-KEY (obesity) OR TITLE-ABS-KEY (hyperlipidemia) OR TITLE-ABS-KEY (abdominal obesity)	25
	KS-3: (TITLE-ABS-KEY (curcumin) OR TITLE-ABS-KEY (diferuloylmethane) OR TITLE-ABS-KEY AND TITLE-ABS-KEY ("in vivo") OR TITLE-ABS-KEY (rat) OR TITLE-ABS-KEY (mice) AND TITLE-ABS-KEY (metabolic syndromes) AND TITLE-ABS-KEY (hypertension) OR TITLE-ABS-KEY (high blood pressure) OR TITLE-ABS-KEY (cardiovascular diseases)	53
	KS-4: (TITLE-ABS-KEY (curcumin) OR TITLE-ABS-KEY (diferuloylmethane) OR TITLE-ABS-KEY AND TITLE-ABS-KEY ("in vivo") OR TITLE-ABS-KEY (rat) OR TITLE-ABS-KEY (mice) AND TITLE-ABS-KEY (metabolic syndromes) OR TITLE-ABS-KEY (heart diseases) OR TITLE-ABS-KEY (stroke)	13
	KS-5: (TITLE-ABS-KEY (curcumin) OR TITLE-ABS-KEY (diferuloylmethane) OR TITLE-ABS-KEY AND TITLE-ABS-KEY ("in vivo") OR TITLE-ABS-KEY (rat) OR TITLE-ABS-KEY (mice) AND TITLE-ABS-KEY (metabolic syndromes) OR TITLE-ABS-KEY (abnormal cholesterol levels) OR TITLE-ABS-KEY (cholesterol profile) OR TITLE-ABS-KEY (hypercholesterolemia)	18

Database	Keywords	Results

AMED 03/10/2024	KA-1: TITLE curcumin or diferuloylmethane AND AB (mice or rat) AND AB metabolic syndromes AND AB insulin resistance OR AB hyperglycemia AND AB type 2 diabetes	29
	KA-2: TITLE curcumin or diferuloylmethane AND AB (mice or rat) AND AB metabolic syndromes AND AB obesity OR AB abdominal obesity AND AB hyperlipidemia	29
	KA-3: TITLE curcumin or diferuloylmethane AND AB mice or rat AND AB metabolic syndromes OR AB hypertension OR AB high blood pressure OR AB cardiovascular diseases	29
	KA-4: TITLE curcumin or diferuloylmethane AND AB mice or rat or vivo AND AB metabolic syndromes OR AB heart diseases OR AB stroke	29
	KA-5: TITLE curcumin or diferuloylmethane AND AB mice or rat or vivo AND AB metabolic syndromes AND AB abnormal cholesterol levels OR AB cholesterol profile OR AB hypercholesterolemia	29
LILACS (2024.04.04)	KL-1: Title, abstract, subject: curcumin or diferuloylmethane and rat or mice and metabolic syndromes and insulin resistance or type 2 diabetes or hyperglycemia	8
	KL-2: Title, abstract, subject: curcumin or diferuloylmethane and rat or mice and metabolic syndromes and obesity or hyperlipidemia or abdominal obesity	8
	KL-3: Title, abstract, subject: curcumin or diferuloylmethane and rat or mice and metabolic syndromes and hypertension or high blood pressure or cardiovascular diseases	8
	KL-4: Title, abstract, subject: curcumin or diferuloylmethane and rat or mice and metabolic syndromes and heart diseases or stroke	7
	KL-5: Title, abstract, subject: curcumin or diferuloylmethane and rat or mice and metabolic syndromes or abnormal cholesterol levels or cholesterol profile or hypercholesterolemia	8

Database	Keywords	Results
MDPI	KM-1: Title: curcumin or diferuloylmethane or *Curcuma longa* and Abstract : vivo or rat or mice and metabolic syndromes and insulin resistance and type 2 diabetes	20
	KM-2: Title: curcumin or diferuloylmethane or *Curcuma longa* and Abstract : rat or mice and metabolic syndromes and obesity or abdominal obesity and hyperlipidemia	11
	KM-3: Title: curcumin or diferuloylmethane or *Curcuma longa* and Abstract : rat or mice and metabolic syndromes and hypertension or high blood pressure and cardiovascular diseases	88
	KM-4: Title: curcumin or diferuloylmethane or *Curcuma longa* and Abstract : rat or mice and metabolic syndromes and heart diseases and stroke	1
	KM-5: Title: curcumin or diferuloylmethane or *Curcuma longa* and Abstract : rat or mice and metabolic syndromes and abnormal cholesterol levels and hypercholesterolemia or cholesterol profile	12
Google Scholar	Only English and Relevant articles are deducted. Others unnecessary and uninterested are removed and excluded.; Main keywords- Curcumin, rat or mice or vivo; Exclude the words - review, meta-analysis, clinical, human, men, women, child, children, hospital. Method - Title Screening Additional Keywords: Insulin Resistance, Type-2-diabetes, Hyperglycemia, Metabolic syndrome, Obesity, Hyperlipidemia, Hypertension, Stroke, Heart disease, Hypercholesterolemia, cholesterol	41

Supplementary Table S2. Assessment of methodological quality in the studies included using the 10-item CAMARADES check-list

	Checklist items									
Author, Year	1	2	3	4	5	6	7	8	9	10
Abiodun et al. [24]	y	n	y	y	n	y	y	n	y	n
Afifi et al. [25]	y	n	y	y	n	y	y	n	y	n
Ahmed et al. [26]	y	y	n	y	n	y	y	n	y	y
Akintunde et al. [27]	y	y	y	y	n	y	y	n	y	n
Amin et al. [28]	y	y	n	y	n	y	y	n	y	n
Ariamoghaddam et al. [29]	y	n	n	y	n	y	y	n	y	n
Auger et al. [30]	y	n	y	y	n	y	y	n	y	y
Bulboacă et al. [31]	y	y	y	y	n	y	y	n	y	y
D'Antongiovanni et al. [32]	y	y	y	y	n	y	y	n	y	y
Demir [33]	y	y	y	y	n	y	y	n	y	y
Ding et al. [34]	y	y	y	y	n	y	y	n	y	y
Ding et al. [35]	y	n	n	y	n	y	y	n	y	y
Eissa et al. [36]	y	n	n	y	n	y	y	n	y	n
Ejaz et al. [37]	y	n	y	y	n	y	y	n	y	n
Hong et al. [38]	y	n	y	y	n	y	y	n	y	y
Hu et al. [39]	y	n	y	y	n	y	y	n	y	n
Hu et al. [40]	y	n	n	y	n	y	y	n	y	n
Hussein et al. [41]	y	y	n	y	n	y	y	n	y	n
Ibrahim et al. [42]	y	y	n	y	n	y	y	n	y	y
Kapar et al. [43]	y	y	n	y	n	y	y	n	y	y
Kelany et al. [44]	y	y	n	y	n	y	y	n	y	y
Kobori et al. [45]	y	y	n	y	n	y	y	n	y	y
Koboziev et al. [46]	y	y	y	y	n	y	y	n	y	y
Lee et al. [47]	y	y	y	y	n	y	y	n	y	y
Li et al. [48]	y	y	y	y	n	y	y	n	y	n
Li et al. [49]	y	y	y	y	n	y	y	n	y	y
Li et al. [50]	y	y	y	y	n	y	y	n	y	y
Li et al. [51]	y	y	y	y	n	y	y	n	y	y
Majithiya et al. [52]	y	y	n	y	n	y	y	n	y	n
Miyazawa et al. [53]	y	n	n	y	n	y	y	n	y	y
Mohammadi et al. [54]	y	n	n	y	n	y	y	n	y	y
Neyrinck et al. [55]	y	n	n	y	n	y	y	n	y	y
Omaima & Fouad [56]	y	n	n	y	n	y	y	n	y	n
Pan et al. [57]	y	y	y	y	n	y	y	n	y	y

Preez et al. [58]	y	y	y	y	n	y	y	n	y	y
Ramesh et al. [59]	y	n	n	y	n	y	y	n	y	n
Rao et al. [60]	y	n	n	y	n	y	y	n	y	n
Rivergo-Salgado et al. [61]	y	y	y	y	n	y	y	n	y	y
Samadder et al. [62]	y	y	n	y	n	y	y	n	y	y
Sarker et al. [63]	y	n	y	y	n	y	y	n	y	n
Severcan et al. [64]	y	y	n	y	n	y	y	n	y	y
Su et al. [65]	y	n	y	y	n	y	y	n	y	n
Tiwari-Pandey et al. [66]	y	n	n	y	y	y	y	n	y	n
Wu et al. [67]	y	n	y	y	n	y	y	n	y	n
Zhang et al. [68]	y	n	y	y	n	y	y	n	y	n
Zhong et al. [69]	y	y	y	y	n	y	y	n	y	y
Zou et al. [70]	y	y	y	y	n	y	y	n	y	y

Studies fulfilling the criteria of: 1= the peer-reviewed publication; 2=control of temperature; 3=random allocation to treatment or control; 4=blinded induction of metabolic syndrome; 5=blinded assessment of outcome; 6=use of co-interventions/co-morbid without significant intrinsic protective effect toward metabolic syndromes; 7=appropriate animal model (age, sex, species, strain); 8=sample size calculation; 9=compliance with animal welfare regulations; and 10=statement of potential conflict of interests.

Table S3. Risk of bias assessment of the included studies using the SYRCLE tool.

Author Year	Types of bias and check items									
	Selection bias			Performance bias		Detection bias		Attrition bias	Reporting bias	Other sources of bias
	SB1	SB2	SB3	PB1	PB2	DB1	DB2			
Abiodun et al. [24]	High	Unclear	Low	Unclear	Low	Low	Low	Unclear	Unclear	High
Afifi et al. [25]	High	Unclear	Low	Unclear	Low	Low	Low	Low	Unclear	High
Ahmed et al. [26]	Low	High	Low	Unclear	Low	Low	Low	Unclear	Unclear	High
Akintunde et al. [27]	High	Unclear	Low	Unclear	Low	Low	Low	Low	High	High
Amin et al. [28]	Low	High	Low	Unclear	Low	Low	Low	Low	High	High
Ariamoghaddam et al. [29]	Low	Unclear	Low	Low	Low	Low	Low	Unclear	Unclear	High
Auger et al. [30]	High	High	Low	Unclear	Low	Low	Low	Low	Unclear	High
Bulboacă et al. [31]	High	Unclear	Low	Unclear	Low	Low	Low	Unclear	Unclear	High
D'Antongiovanni et al. [32]	High	High	Low	Unclear	Low	Low	High	Low	Unclear	High
Demir [33]	High	Unclear	Low	Unclear	Low	Low	Low	Low	High	High
Ding et al. [34]	High	High	Low	Unclear	Low	Low	Low	Unclear	Unclear	High
Ding et al. [35]	Low	High	Low	Low	Low	Low	Low	Unclear	Unclear	High
Eissa et al. [36]	Low	Unclear	Low	Low	Low	Low	Low	Low	High	High
Ejaz et al. [37]	High	High	Low	Low	Low	Low	Low	Low	High	High
Hong et al. [38]	High	Unclear	Low	Unclear	Low	Low	Low	Low	High	High
Hu et al. [39]	High	Unclear	Low	Low	Low	Low	Low	Low	High	High
Hu et al. [40]	Low	Low	Low	Unclear	Low	Low	Low	Low	High	High
Hussein et al. [41]	Low	High	Low	Low	Low	Low	Low	Low	Unclear	High
Ibrahim et al. [42]	Low	High	Low	Unclear	Low	Low	Low	Low	Unclear	High
Kapar et al. [43]	Low	Unclear	Low	Low	Low	Low	Low	Low	Unclear	High
Kelany et al. [44]	Low	High	Low	Unclear	Low	Low	Low	Low	High	High
Kobori et al. [45]	Low	High	Low	Unclear	Low	Low	Low	Low	Unclear	High
Koboziev et al. [46]	High	High	Low	Unclear	Low	Low	Low	Low	Unclear	High

Lee et al. [47]	High	High	Low	Unclear	Low	Low	Low	Unclear	Unclear	High	28
Li et al. [48]	High	High	Low	Unclear	Low	Low	Low	Low	High	High	29
Li et al. [49]	High	Unclear	Low	Low	Low	Low	Low	Low	High	High	30
Li et al. [50]	High	High	Low	Unclear	Low	Low	Low	Low	High	High	31
Li et al. [51]	High	Low	Low	Unclear	Low	Low	Low	Low	Unclear	High	32
Majithiya et al. [52]	Low	Unclear	Low	Unclear	Low	Low	Low	Low	High	High	33
Miyazawa et al. [53]	Low	High	Low	Unclear	Low	Low	Low	Unclear	Unclear	High	34
Mohammadi et al. [54]	Low	Low	Low	Low	Low	Low	Low	Low	High	High	35
Neyrinck et al. [55]	Low	Unclear	Low	Unclear	Low	Low	Low	Low	High	High	36
Omaima & Fouad [56]	Low	Unclear	Low	Unclear	Low	Low	Low	Unclear	Unclear	High	37
Pan et al. [57]	High	High	Low	Low	Low	Low	Low	Low	Unclear	High	38
Preez et al. [58]	High	High	Low	Unclear	Low	Low	Low	Low	Unclear	Unclear	39
Ramesh et al. [59]	Low	Unclear	Low	Unclear	Low	Low	Low	Low	High	High	40
Rao et al. [60]	Low	Unclear	Low	Unclear	Low	Low	Low	Unclear	Unclear	High	41
Rivergo-Salgado et al. [61]	High	High	Low	Unclear	Low	Low	Low	Low	High	High	42
Samadder et al. [62]	Low	Unclear	Low	Unclear	Low	Low	Low	Unclear	Unclear	High	43
Sarker et al. [63]	High	High	Low	Low	Low	Low	Low	Unclear	Unclear	High	44
Severcan et al. [64]	Low	Unclear	Low	Unclear	Low	Low	Low	Low	High	High	45
Su et al. [65]	High	Unclear	Low	Low	Low	Low	Low	Low	High	High	46
Tiwari-Pandey et al. [66]	Low	Low	Low	Unclear	Low	High	Low	Low	Unclear	High	47
Wu et al. [67]	High	High	Low	Unclear	Low	Low	Low	Low	Unclear	High	48
Zhang et al. [68]	High	Unclear	Low	Unclear	Low	Low	Low	Low	Unclear	High	49
Zhong et al. [69]	High	High	Low	Unclear	Low	Low	Low	Low	Unclear	High	50
Zou et al. [70]	High	Unclear	Low	Unclear	Low	Low	Low	Low	High	High	51

52

Supplementary Table S4. Strategies for inducing metabolic syndrome in animal models for included studies.

Author, Year	Methods used	The construction of the induction			
		Diet	Chemical	Genetic	Other
Abiodun et al. [24]	HFD	√	–	–	–
Afifi et al. [25]	10% Fructose solution (drinking water)	√	–	–	–
Ahmed et al. [26]	10% fructose solution (drinking water) and 3% NaCl with food pellets	√	–	–	–
Akintunde et al. [27]	Single daily dose of Bisphenol A (BPA) (50mg/kg) dissolved with olive oil	–	√	–	–
Amin et al. [28]	60-66% fructose in diet	√	–	–	–
Ariamoghaddam et al. [29]	High-calorie diet by adding fructose to their drinking water for 4 months	√	–	–	–
Auger et al. [30]	Risperdal (risperidone) with intraperitoneal injection at a dose of 12.5 mg per kg	–	√	–	–
Bulboacă et al. [31]	Fructose 10g in 100ml tap water + Streptozotocin-induced diabetes mellitus intraperitoneally 60mg/kg	√	√	–	–
D'Antongiovanni et al. [32]	High-fat diet (HFD)	√	–	–	–
Demir [33]	A single intraperitoneal injection of STZ (60 mg/kg)	–	√	–	–
Ding et al. [34]	10% fructose solution (water)	√	–	–	–
Ding et al. [35]	High-fat diet (HFD)	√	–	–	–
Eissa et al. [36]	High carbohydrate High fat (HCHF) (50% carbohydrate, 24% fat) diet (16 weeks)	√	–	–	–
Ejaz et al. [37]	AIN-93 diet containing 22% fat by weight	√	–	–	–
Hong et al. [38]	0.5mg/kg Bisphenol A(BPA) incorporated into the normal diet	–	√	–	–
Hu et al. [39]	High-fat diet (HFD)	√	–	–	–
Hu et al. [40]	Spontaneous hypertensive rats (SHRs)	–	–	√	–
Hussein et al. [41]	High-fat diet (HFD)	√	–	–	–
Ibrahim et al. [42]	Fructose diet (20%, w/v) induced	√	–	–	–
Kapar et al. [43]	Fresh fructose at a rate of 20% and given as ad libitum daily for eight weeks	√	–	–	–
Kelany et al. [44]	High-fat diet (HFD)	√	–	–	–
Kobori et al. [45]	Western-style modified	√	–	–	–

	AIN93G diet					
Koboziew et al. [46]	Very high fat diet (60% kcal fat, 20% kcal carbohydrate, and 20% kcal protein)	√	–	–	–	
Lee et al. [47]	High-fat diet (HFD)	√	–	–	–	
Li et al. [48]	High-fat diet (HFD)	√	–	–	–	
Li et al. [49]	Spontaneous hypertensive rats (SHRs)	–	–	√	–	
Li et al. [50]	High-fat diet (HFD)	√	–	–	–	
Li et al. [51]	High-fat diet (12 weeks) + intraperitoneal injection of 2ml/kg Vitamin D3 daily for 3 days	√	√	–	–	
Majithiya et al. [52]	Injection of 200mg/kg of triton WR 1339 in normal saline	–	√	–	–	
Miyazawa et al. [53]	High-fat diet (HFD)	√	–	–	–	
Mohammadi et al. [54]	Polycystic ovary Syndrome (PCOS) rats	–	–	–	√	
Neyrinck et al. [55]	ob/ob mice (B6.V-Lepob/ob/JRj)	–	–	√	–	
Omaima & Fouad [56]	High Fat Diet (2 weeks) + intraperitoneal injection of STZ (50mg/kg) dissolved in 0.2ml of citrate buffer		√	√	–	–
Pan et al. [57]	High-fat diet (HFD)	√	–	–	–	
Preez et al. [58]	High carbohydrate, high-fat diet (simple sugars and both saturated and trans fats)	√	–	–	–	
Ramesh et al. [59]	High-fat diet (HFD)+Olanzapine	√	√	–	–	
Rao et al. [60]	Cholesterol supplemented diet	√	–	–	–	
Rivego-Sagado et al. [61]	Hypercaloric diet (Carbohydrates: 49.3%, Lipids: 21.0%, Protein: 19.5%, Fiber: 5.0%, Minerals: 3.5% Vitamins: 1.7%, Energy content: 19.43 kJ/g)	√	–	–	–	
Samadder et al. [62]	Alloxan monohydrate (ALX) dissolved in sterile normal saline was injected at a dose of 100 mg/kg body weight	–	√	–	–	
Sarker et al. [63]	Standard mice pellet enriched with 10%butter feeding	√	–	–	–	
Severcan et al. [64]	20 % D-fructose prepared with tap water	√	–	–	–	
Su et al. [65]	High fat and glucose diet (4 weeks) + intraperitoneal injection of streptozotocin (1%, 25mg/kg) prepared by the citric acid and sodium citrate buffer	√	√	–	–	

Tiwari-Pandey et al. [66]	High-fat diet (HFD)	√	–	√	–	128
Wu et al. [67]	6 weekly injections of azoxymethane (5mg/kg intraperitoneally at the first injection and 10mg/kg intraperitoneally, high-fat diet induction	√	√	–	–	129 130 131 132
Zhang et al. [68]	10% fructose solution (drinking water)	√	–	–	–	133
Zhong et al. [69]	High-fat diet (HFD)	√	–	–	–	134
Zou et al. [70]	High-fat diet (HFD)	√	–	√	–	135

136

Supplementary Table S5. Environmental/Feeding conditions of animal model included in the studies.

Author, Year	Temperature °C	Light/dark cycle	Humidity	Timing of feed Administration
Abiodun et al. [24]	N/M	N/M	N/M	N/M
Afifi et al. [25]	N/M	N/M	N/M	N/M
Ahmed et al. [26]	22 ± 3	12:12 hour light-dark cycle	50-60%	N/M
Akintunde et al. [27]	25 ± 2	12:12 hour light-dark cycle	N/M	ad libitum
Amin et al. [28]	23-25	N/M	N/M	ad libitum
Ariamoghaddam et al. [29]	N/M	N/M	N/M	N/M
Auger et al. [30]	N/M	12:12 hour light-dark cycle	N/M	ad libitum
Bulboacă et al. [31]	24±2	12:12 hour light-dark cycle	60±5%	ad libitum
D' Antongiovanni et al. [32]	22–24	12:12 hour light-dark cycle	50–60%	N/M
Demir [33]	22 ± 2	12:12 hour light-dark cycle	55 ± 5%	N/M
Ding et al. [34]	N/M	12:12 hour light-dark cycle	N/M	N/M
Ding et al. [35]	N/M	12:12 hour light-dark cycle	N/M	N/M
Eissa et al. [36]	N/M	N/M	N/M	ad libitum
Ejaz et al. [37]	N/M	N/M	N/M	N/M
Hong et al. [38]	N/M	N/M	N/M	N/M
Hu et al. [39]	N/M	N/M	N/M	N/M
Hu et al. [40]	N/M	N/M	N/M	N/M
Hussein et al. [41]	25	Natural light-dark	60-70%	ad libitum
Ibrahim et al. [42]	N/M	N/M	N/M	N/M
Kapar et al. [43]	22 ± 2	12:12 hour light-dark cycle	60%	ad libitum
Kelany et al. [44]	22 ± 2	12:12 hour light-dark cycle	N/M	ad libitum
Kobori et al. [45]	24 ± 1	12:12 hour light-dark cycle	55% ± 5%	ad libitum
Koboziew et al. [46]	23	N/M	N/M	N/M
Lee et al. [47]	22 - 25	12:12 hour light-dark cycle	N/M	N/M
Li et al. [48]	24 ± 1	12:12 hour light-dark cycle	55 ± 5%	N/M
Li et al. [49]	22	12:12 hour light-dark cycle	N/M	N/M
Li et al. [50]	23 ± 2	12:12 hour light-dark cycle	N/M	ad libitum
Li et al. [51]	22	12:12 hour light-dark cycle	N/M	ad libitum
Majithiya et al. [52]	24	12:12 hour light-dark cycle	N/M	ad libitum
Miyazawa et al. [53]	N/M	12:12 hour light-dark cycle	N/M	N/M
Mohammadi et al. [54]	N/M	12:12 hour light-dark cycle	N/M	N/M
Neyrinck et al. [55]	N/M	12:12 hour light-dark cycle	N/M	ad libitum
Omaima & Fouad [56]	N/M	N/M	N/M	N/M
Pan et al. [57]	22 ± 1	12:12 hour light-dark cycle	50%	ad libitum
Preez et al. [58]	21 ± 2	12:12 hour light-dark cycle	N/M	ad libitum
Ramesh et al. [59]	N/M	12:12 hour light-dark cycle	N/M	ad libitum
Rao et al. [60]	N/M	N/M	N/M	N/M
Rivego-Sagado et al. [61]	22 ± 1	12:12 hour light-dark cycle	55 ± 5%	ad libitum
Samadder et al. [62]	24-26 ± 2	12:12 hour light-dark cycle	55 ± 5%	ad libitum
Sarker et al. [63]	N/M	N/M	N/M	N/M
Severcan et al. [64]	22 ± 2	12:12 hour light-dark cycle	N/M	ad libitum

Su et al. [65]	N/M	N/M	N/M	N/M
Tiwari-Pandey et al. [66]	N/M	N/M	N/M	ad libitum
Wu et al. [67]	N/M	N/M	N/M	N/M
Zhang et al. [68]	N/M	12:12 hour light-dark cycle	N/M	N/M
Zhong et al. [69]	23	12:12 hour light-dark cycle	N/M	ad libitum
Zou et al. [70]	22°C ± 2	12-hour light/dark cycle	N/M	N/M

Supplementary Table S6. The summary of outcomes related to parameters associated with metabolic syndrome, inflammation, and oxidative stress highlights the effects of curcumin/*Curcuma longa* extracts in rodent models.

Author, Year	Metabolic syndrome related parameters	Inflammatory markers	Oxidative stress markers
Abiodun et al. [24]	↓TC, ↓TG, ↓LDL, ↑HDL	-	-
Afifi et al. [25]	↓WG, ↓Fat, ↓TG, ↓Glu, ↓INS, ↓TC, ↓LDL, ↑HDL	↓IL-6, ↓CRP, ↓TNF-α	↓MDA, ↑SOD
Ahmed et al. [26]	-	-	-
Akintunde et al. [27]	-	↓NO	↓MDA, ↑CAT, ↑SOD
Amin et al. [28]	↓TG, ↓Glu, ↓INS, ↓TC, ↓LDL, ↑HDL	↓CRP	-
Ariamoghaddam et al.[29]	↓WG, ↓TG, ↓TC	-	-
Auger et al. [30]	↓WG, ↓TG, ↓Glu, ↓TC, ↓HDL	↓TNF-α, ↓NFκB	-
Bulboacă et al. [31]	↓TG, ↓Glu, ↓TC, ↓LDL, ↑HDL, ↓BP	↓NOx	↓MDA, ↓TOS, ↑TAC, ↑Thiol
D'Antongiovanni et al.[32]	↓TG, ↓TC, ↓Hb1Ac, ↓BMI	↓IL-1β, ↓MPO	-
Demir [33]	↓TC	-	↓MDA, ↑GSH, ↑CAT,↑GST
Ding et al. [34]	↓WG, ↑TG, ↓Glu, ↓INS, ↓TC	-	-
Ding et al. [35]	↓WG, ↓Fat, ↓TG, ↓Glu, ↓INS, ↓TC, ↓LDL, HDL,	-	-
Eissa et al. [36]	↓TG, ↓Glu, ↓TC, ↓LDL, ↑HDL, ↓BP	-	↓MDA
Ejaz et al., [37]	↓WG, ↓Fat, ↓TG, ↓Glu, ↓TC	-	-
Hong et al. [38]	↓WG, ↓TG, ↓TC, ↓LDL,	-	-
Hu et al. [39]	↓TG, ↓Glu, ↓TC, ↓LDL	-	-
Hu et al. [40]	↓BP	-	-
Hussein et al. [41]	↓WG, ↓TG, ↓Glu, ↓TC, ↓LDL, ↑HDL	-	↓MDA
Ibrahim e al. [42]	↑Glu, INS	-	-
Kapar et al. [43]	↓TG, ↓Glu, ↓INS, ↓TC	↓NO	-

Kelany et al. [44]	↓WG, ↓TG, ↓Glu, ↓INS, ↓TC, ↓BP	↓TNF-α	↓MDA	192 193
Kobori et al. [45]	↓WG, ↓Fat, ↓TG, ↓BP, ↓INS, ↑TC	↓TNF-α, ↓INF-γ	↓MDA	194 195
Koboziew et al. [46]	↓WG, ↓Fat	-	-	196
Lee et al. [47]	↓ WG, ↓ TG, ↓ Glu, ↓ INS, ↓ TC-	-		197
Li et al. [48]	↓TG, ↓TC, ↓LDL, ↑HDL	-	-	198
Li et al. [49]	BP ↓	↓TNF-α, ↓MMP-9	-	199
Li et al. [50]	↓WG, Glu, ↓INS, ↓NAFLD	-	-	200
Li et al. [51]	TG, ↓TC, ↓LDL, ↑HDL	-	-	201
Majithiya et al. [52]	↑TG, ↓TC	-	-	202
Miyazawa et al. [53]	WG, Fat	↓IL-6, ↓INF-γ, ↓IL-10, ↓IL-12, ↓IL-1β	-	203 204
Mohammadi et al. [54]	↓Glu, ↓INS	↓IL-6, ↓CRP	-	205
Neyrinck et al. [55]	↓WG, Fat, TG, ↓Glu, ↓INS, TC	↑IL-6, ↑TNF-α	-	206
Omaima & Fouad, [56]	↓Glu, ↓INS	↓IL-6, ↓TNF-α, ↓IL-1β	-	207
Pan et al. [57]	↓WG, ↓Fat, ↓TG, ↓Glu, ↓INS -		-	208
Preez et al. [58]	↓WG, ↓Fat, ↓TG, ↓Glu, ↓TC, BP, -		-	209 210
Ramesh et al. [59]	↓WG, ↓TG, ↓Glu, ↓TC, ↓LDL, ↑HDL -		-	211 212
Rao et al. [60]	↓TC -		-	213
Rivego-Sagado et al. [61]	WG, ↑TG, ↓Glu, INS, TC -		-	214
Samadder et al. [62]	↓Glu -		-	215
Sarker et al. [63]	↓WG, ↓TG, ↓TC, ↓LDL, ↑HDL -		-	216 217
Severcan et al. [64]	TG, ↓Glu, ↓INS, TC, LDL, HDL, ↓BP	↓TNF-α	↓TOS, ↑TAC	218 219
Su et al. [65]	↓Glu, ↓TC, ↓LDL, ↓HDL	↓TNF-α	-	220
Tiwari-Pandey et al. [66]	↓WG, ↓Fat -		-	221
Wu et al. [67]	↓WG, ↓Fat	↓IL-6	-	222
Zhang et al. [68]	↓WG, ↓TG, ↓TC, ↓LDL, ↑HDL, ↓BP	↑NO	-	223 224
Zhong et al. [69]	↓Glu	-	-	225
Zou et al. [70]	↓WG, ↓TC, ↓LDL, ↑HDL	-	-	226

Supplementary Table S7. Evaluating the effects of curcumin/*Curcuma longa* extracts consumption on weight gain (WG), BMI, insulin (INS), glucose (Glu), HbA1c, and blood pressure (BP) in rodent models of metabolic syndrome.

Author, Year	Measured parameters (mean ± SD)					
(tested groups and doses)	WG (g)	BMI (g/cm^2)	INS	Glu	HbA1c	BP (mmHg)
Afifi et al. [25]						
Control	311.4±14.5	-	8.8±2.5 mM/L	8.8 mM/L	-	-
Curcumin 40 mg/kg	270.0±12.4	-	7.2±1.0 mM/L	7.2 mM/L	-	-
Curcumin 80 mg/kg	265.3±7.0	-	6.0±1.0 mM/L	6.0 mM/L	-	-
Amin et al. [28]						
Control	-	-	17.1±2.3 mg/dl	168.0±5.71 mg/dl	-	-
Turmeric 1.5 g/kg	-	-	12.0±4.1 mg/dl	163.0±8.2 mg/dl	-	-
Turmeric 3g/kg	-	-	7.0±3.7 mg/dl	151.0±10.2 mg/dl	-	-
Ariamoghaddam et al. [29]						
Control	45.7±9.5	-	-	-	-	-
Curcumin	8.6±5.0	-	-	-	-	-
Auger et al. [30]						
Control	14.5±5.5	-	-	180.0±6.0 mg/dl	-	-
Curcumin	13.8±3.0	-	-	150.0±6.0 mg/dl	-	-
Bulboacă et al. [31]						

Control (Fructose)	-	-	-	170.0±1.0 mg/dl	-	178.0±1.0	248
Curcumin 1 g/kg	-	-	-	120.0±1.0 mg/dl	-	165.0±1.5	249
Control (Streptozotocin)	-	-	-	450.0±5.0 mg/dl	-	142.0±1.5	250
D' Antongiovanni et al. [32]							251
Control	-	0.4	-	-	33.9±0.5 mM/M	-	252
Curcumin 49 mg/kg	-	0.4	-	-	28.5±0.5 mM/M	-	253
Ding et al. [34]							254
Control	610.5±18.3	-	0.9±0.1 ng/mL	112.0±2.0 mg/dl	-	-	255
Curcumin 15 mg/kg	66.2±-6.9	-	0.6±0.1 ng/mL	105.0±1.5 mg/dl	-	-	256
Curcumin 30 mg/kg	56.2±-1.7	-	0.4±0.0 ng/mL	106.0±1.8 mg/dl	-	-	257
Curcumin 60 mg/kg	68.67±-1.3	-	0.6±0.1 ng/mL	110.0±1.5 mg/dl	-	-	258
Pioglitazone	542.4±12.1	-	0.4±0.1 ng/mL	104.0±1.5 mg/dl	-	-	259
Ding et al. [35]							260
Control	30.0	-	3.5±4.0 ng/mL	8.7±2.5 mM/L	-	-	261
Curcumin 40 mg/kg	25.7	-	3.1±4.5 ng/mL	8.2±2.5 mM/L	-	-	262
Curcumin 80 mg/kg	21.4	-	2.0±3.0 ng/mL	6.0±2.0 mM/L	-	-	263
Lovastatin	24.3	-	3.3±2.0 ng/mL	7.6±3.0 mM/L	-	-	264
							265
							266
							267
Eissa et al. [36]							268

Control	-	-	-	7.8±1.6 mM/L	-	142.5±9.2	269
Curcumin 200 mg/kg	-	-	-	6.1±1.2 mM/L	-	117.7±4.8	270
Atorvastatin	-	-	-	6.0±1.5 mM/L	-	119.5±9.3	271
Ejaz et al. [37]							272
Control	37.0±2.9	-	-	16.4±2.1 mM/L	-	-	273
Curcumin 500 mg/kg	33.1±2.1	-	-	15.4±1.5 mM/L	-	-	274
Hong et al. [38]							275
Control	41.5±7.6	-	-	-	-	-	276
Curcumin 0.1%	39.1±3.8	-	-	-	-	-	277
Hu et al. [39]							278
Control	-	-	-	14.3±1.0 mM/L	-	-	279
Curcumin 200 mg/kg	-	-	-	8.3±1.5 mM/L	-	-	280
Hu et al. [40]							281
Control	-	-	-	-	-	193.2±1.4	282
Curcumin 25 mg/kg	-	-	-	-	-	189.3±2.3	283
Curcumin 50 mg/kg	-	-	-	-	-	189.8±0.7	284
Curcumin 100 mg/kg	-	-	-	-	-	191.0±1.6	285
Curcumin 200 mg/kg	-	-	-	-	-	191.5±1.3	286
Curcumin 400 mg/kg	-	-	-	-	-	190.5±1.5	287
Hussein et al. [41]							288

	Control	207.3±1.0	-	-	180.0±0.2 mg/dl	-	-
	Curcumin 80 mg/kg	116.4±0.5	-	-	120.0±1.0 mg/dl	-	-
Ibrahim e al. [42]							
Male	Control (Fructose)	-	-	0.1±0.0 nM/L	4.0±0.1 mM/L	-	-
	Curcumin 500 mg/kg	-	-	0.1±0.0 nM/L	4.4±0.2 mM/L	-	-
Female	Control (Fructose)	-	-	0.1±0.0 nM/L	4.0±0.1 mM/L	-	-
	Curcumin 500 mg/kg	-	-	0.1±0.0 nM/L	4.0±0.1 mM/L	-	-
Kapar et al. [43]							
	Control	-	-	10.5±0.4 Mu/mL	280.5±18.6 mg/dl	-	-
	Curcumin 100 mg/kg	-	-	6.3±1.2 Mu/mL	198.6±11.0 mg/dl	-	-
Kelany et al. [44]							
	Control	90.1±9.8	-	45.2±4.0 µU/mL	185.2±15.1 mg/dl	-	157.3±14.0
	Curcumin 200 mg/kg	26.3±3.8	-	34.5±1.9 µU/mL	130.2±3.4 mg/dl	-	134.1±9.51
Kobori et al. [45]							
	Control	6.0±0.8	-	4.0±0.7 ng/mL	-	-	-
	Curcumin 0.1%	3.4±0.8	-	3.1±0.4 ng/mL	-	-	-
Koboziew et al. [46]							
	Control	10.7±2.5	-	-	-	-	-
	Curcumin 0.7%	12.9±3.0	-	-	-	-	-
Lee et al. [47]							
	Control	23.2	-	0.5±5.0 mg/dl	200.0±2.0 mg/dl	-	-

Curcumin	19.0	-	0.4±2.5 mg/dl	200.0±2.5 mg/dl	-	-	312
Curcumin5-8	21.5	-	0.3±3.5 mg/dl	201.0±2.0 mg/dl	-	-	313
Li et al. [48]							314
Control	17.8±3.0	-	36.7±3.0 mU/L	10.0±1.5 mM/L	-	-	315
Curcumin 2000 mg/kg	13.3±2.0	-	25.0±1.5 mU/L	7.6±2.5 mM/L	-	-	316
Li et al. [49]							317
Control	-	-	-	-	-	93.3±2.5	318
Curcumin 100 mg/kg	-	-	-	-	-	57.8±2.5	319
Curcumin 100 mg/kg	-	-	-	-	-	51.1±3.0	320
Miyazawa et al. [53]							321
Control	23	-	-	-	-	-	322
Curcumin 1 g/kg	24	-	-	-	-	-	323
Mohammadi et al. [54]							324
Control	-	-	33.0±0.1 U	25.0±0.1 mm/L	-	-	325
Curcumin 100 mg/kg	-	-	27.0±0.1 U	22.0±0.1 mm/L	-	-	326
Curcumin 300 mg/kg	-	-	13.0±0.1 U	17.0±0.1 mm/L	-	-	327
Neyrinck et al. [55]							328
Control	5.4±0.3	-	13.5±4.0 µg/L	153.9±3.5 mg/dl	-	-	329
Curcumin 0.3%	4.8±0.2	-	13.5±4.0 µg/L	169.2±3.5 mg/dl	-	-	330
							331
Omaima and Fouad, [56]							332
Curcumin 200 mg/kg							333
Pre-treatment	-	-	5.1±1.3 IU/mL	259.9±35.7 mg/dl	-	-	334

Post-treatment	-	-	3.6±0.4 IU/mL	160.7±18.1 mg/dl	-	-	335
Pan et al., [57]							336
Control	9.0±0.4 g	-	63.3±2.5 mU/L	8.3±0.8 mM/L	-	-	337
THC 20 mg/kg	5.0±2.7 g	-	10.8±0.5 mU/L	7.6±1.0 mM/L	-	-	338
THC 100 mg/kg	5.7±3.3 g	-	10.8±1.5 mU/L	6.9±2.0 mM/L	-	-	339
Preez et al. [58]							340
Control (HFD)	176.0±10.0	-	-	3.4±0.4 mM/L	-	143.0±5.0	341
Curcumin 5 mg/kg	153.0±9.0	-	-	3.9±0.2 mM/L	-	143.0±4.0	342
Curcumin 100 mg/kg	161.0±9.0	-	-	3.9±0.4 mM/L	-	126.0±4.0	343
CCNP	153.0±5.0	-	-	4.5±0.2 mM/L	-	128.0±3.0	344
Ramesh et al. [59]							345
Control (HFD)	284.5±3.2	-	-	158.3±1.2	-	-	346
Curcumin 50 mg/kg	215.7±3.8	-	-	114.7±1.6	-	-	347
Telmisartan	183.3±2.4	-	-	109.5±3.1	-	-	348
Rivego-Sagado et al. [61]							349
A normal litter; Control	395.0±2.5	-	6.0±1.0 ng/mL	115.0±1.0 mg/dL	-	-	350
A normal litter; Curcumin	375.0±2.0	-	5.6±1.0 ng/mL	110.0±1.5 mg/dL	-	-	351
A small litter; Control	415.0±2.5	-	6.3±1.0 ng/mL	95.0±2.0 mg/dL	-	-	352
A small litter; Curcumin	425.0±2.0	-	7.3±0.5 ng/mL	105.0±0.8 mg/dL	-	-	353
							354
							355
							356
Samadder et al. [62]							357
Control	-	-	-	195.5±1.3 mg/dl	-	-	358
Curcumin 50 mg/kg	-	-	-	116.0±2.2 mg/dl	-	-	359
NCUR1 25 mg/kg	-	-	-	128.0±1.8 mg/dl	-	-	360

NCUR2 21.5 mg/kg	-	-	-	126.0±2.0 mg/dl	-	-	361
Sarker et al. [63]							362
Control	56.1±1.4	-	-	-	-	-	363
Curcumin 1%	51.5±1.5	-	-	-	-	-	364
Curcumin 2%	47.7±1.9	-	-	-	-	-	365
Curcumin 3%	48.7±2.2	-	-	-	-	-	366
Severcan et al. [64]							367
Control	-	-	8.1 µU/mL	212.3 mg/dl	-	173.1	368
Curcumin 100 mg/kg	-	-	5.5 µU/mL	249.3 mg/dl	-	154.7	369
Curcumin 200 mg/kg	-	-	4.5 µU/mL	159.3 mg/dl	-	153.5	370
Su et al. [65]							371
Control	-	-	-	15.9±4.0	-	-	372
Curcumin 250 mg/kg	-	-	-	11.8±4.1	-	-	373
Tiwari-Pandey et al. [66]							374
Control	23.5±0.5	-	-	-	-	-	375
Curcumin 25 mg/kg	23.3±0.7	-	-	-	-	-	376
Wu et al. [67]							377
Control	38.4±0.7	-	-	-	-	-	378
Curcumin 2000 mg/kg	35.2±0.8	-	-	-	-	-	379
							380
							381
Zhang et al. [68]							382
Control	412.4±9.1	-	-	-	-	125.6±2.4	383
Curcumin 15 mg/kg	403.5±11.3	-	-	-	-	116.3±1.2	384
Curcumin 30 mg/kg	385.9±5.3	-	-	-	-	106.2±1.2	385
Curcumin 60 mg/kg	387.6±10.8	-	-	-	-	102.4±1.8	386

Pidgitazone	407.5±16.2	-	-	-	-	109.1±0.8
Zhong et al. [69]						
Control	-	-	-	205±1.0 mg/dl	-	-
Curcumin 100 mg/kg	-	-	-	170±0.5 mg/d	-	-
Zou et al. [70]						
Control	15.1±4.2 g	-	-	-	-	-
Curcumin 1000 mg/kg	9.8±3.4 g	-	-	-	-	-

Supplementary Table S8. Evaluating the effects of curcumin/*Curcuma longa* extracts consumption on fat content, lipid profiles, and NAFLD score in rodent models of metabolic syndrome.

Author, Year	Metabolic-related parameters (mean ± SD)					
(tested group)	Fat	TC (mg/dL)	LDL (mg/dL)	HDL (mg/dL)	TG (mg/dL)	NAFLD Score
Abiodun et al. [24]						

Control	-	4.0±0.5 mM/L	1.2±1.0 mM/L	0.8±1.5 mM/L	2.38±1.0 mM/L	-	409
Curcumin 1.5 g/kg	-	2.4±2.0 mM/L	1.0±4.5 mM/L	1.1±1.5 mM/L	1.1±1.5 mM/L	-	410
Curcumin 2 g/kg	-	2.3±2.5 mM/L	1.0±4.0 mM/L	1.1±2.5 mM/L	1.2±1.8 mM/L	-	411
Curcumin 2.5 g/kg	-	2.2±0.5 mM/L	1.0±4.0 mM/L	1.2±1.5 mM/L	0.9±2.0 mM/L	-	412
Afifi et al. [25]							413
Control	2.0±0.1 g	220.0±2.0	105.8±2.0	32.0±1.5	258.8±3.0	-	414
Curcumin 40 mg/kg	1.8±0.1 g	150.0±0.5	70.0±0.5	36.0±1.0	152.9±2.0	-	415
Curcumin 80 mg/kg	1.5±0.1 g	120.0±1.0	47.0±1.0	36.0±2.0	141.2±1.0	-	416
Amin et al. [28]							417
Control	-	73.0±3.4	20.0±2.2	26.0±4.6	58.0±5.3	-	418
Turmeric 1.5 g/kg	-	56.0±3.7	13.0±2.2	33.0±4.9	43.0±5.1	-	419
Turmeric 3g/kg	-	54.0±5.1	9.0±1.7	30.0±4.8	37.0±3.1	-	420
Ariamoghaddam et al. [29]							421
Control	-	74.0 mg/mL	-	-	78.0 mg/Ml	-	422
Curcumin	-	77.0 mg/mL	-	-	62.0 mg/mL	-	423
Auger et al. [30]							424
Control	-	71.1±5.5	-	44.6±5.0	78.0±7.5	-	425
Curcumin	-	61.9±6.0	-	33.9±3.5	46.0±3.5	-	426
Bulboacă et al. [31]							427
Control (Fructose)	-	88.0±2.0	24.0±2.0	26.0±0.5	205.0±1.5	-	428
Curcumin 1 g/kg	-	72.0±3.5	26.0±3.5	32.0±1.0	90.0±1.5	-	429
Control (Streptozotocin)	-	50.0±2.5	32.0±1.5	24.0±1.0	215.0±1.5	-	430
Curcumin 1 g/kg	-	42.0±2.0	24.0±2.0	23.0±0.8	200.0±1.5	-	431

D'Antongiovanni et al. [32]							432
Control	-	186.4±0.5	-	-	178.6±3.0	-	433
Curcumin 49 mg/kg	-	181.8±0.2	-	-	146.4±2.5	-	434
Demir [33]							435
Control	-	3042.5±79.0 µg/g	-	-	-	-	436
Curcumin 1 mg/kg	-	2421.4±317.4 µg/g	-	-	-	-	437
C60 fullenrence	-	2369.7±290.5 µg/g	-	-	-	-	438
Ding et al. [34]							439
Control	-	109.0±2.4	-	-	193.3±38.9	-	440
Curcumin 15 mg/kg	-	69.5±5.4	-	-	120.7±8.8	-	441
Curcumin 30 mg/kg	-	74.3±2.5	-	-	124.7±12.5	-	442
Curcumin 60 mg/kg	-	63.8±2.5	-	-	117.6±7.3	-	443
Pioglitazone	-	67.1±1.8	-	-	136.7±14.9	-	444
Ding et al. [35]							445
Control	2.5±3.0 g	5.7±1.0 mM/L	0.1±3.0 mM/L	3.9±1.0 mM/L	5.7±3.5 mM/L	-	446
Curcumin 40 mg/kg	-	5.1±1.0 mM/L	0.8±1.0 mM/L	3.9±1.5 mM/L	4.0±3.0 mM/L	-	447
Curcumin 80 mg/kg	1.4±2.5 g	4.4±0.5 mM/L	0.7±2.5 mM/L	3.9±1.0 mM/L	3.3±2.0 mM/L	-	448
Lovastatin	1.8±3.5 g	4.9±1.0 mM/L	0.8±3.5 mM/L	3.8±0.1 mM/L	3.7±3.5 mM/L	-	449
							450
							451
Eissa et al. [36]							452
Control	-	251.2±32.3	178.4±32.6	32.7±9.6	170.8±42.9	-	453
Curcumin 200 mg/kg	-	195.2±40.4	121.6±31.1	37.7±7.7	145.2±33.3	-	454
Atorvastatin	-	152.8±43.8	69.7±14.3	49.7±11.4	125.2±31.4	-	455
Ejaz et al. [37]							456
Control	20.9±1.3 g	5.8±0.2 mM/L	-	-	1.5±0.1 mM/L	-	457

Curcumin 500 mg/kg	10.7±1.9 g	3.5±0.3 mM/L	-	-	0.9±0.1 mM/L	-	458
Hong et al. [38]							459
Control	-	10.0±1.0 mM/L	1.0±2.5 mM/L	4.8±1.0 mM/L	0.9±3.0 mM/L	-	460
Curcumin 0.1%	-	7.2±0.5 mM/L	0.4±1.0 mM/L	6.3±2.0 mM/L	0.5±1.0 mM/L	-	461
Hu et al. [39]							462
Control	-	2.6±2.5 mM/L	0.8±3.0 mM/L	-	1.8±3.5 mM/	-	463
Curcumin 200 mg/kg	-	1.5±2.0 mM/L	0.5±2.0 mM/L	-	0.2±0.5 mM/L	-	464
Hussein et al. [41]							465
Control	-	134.1±0.5	64.61±3.0	31.54±1.5	190.9±3.5	-	466
Curcumin 80 mg/kg	-	102.3±0.5	34.6±3.5	44.62±2.0	106.8±2.0	-	467
Kapar et al. [43]							468
Control	-	41.4±2.2	-	-	218.3±15.5	-	469
Curcumin 100 mg/kg	-	33.9±2.5	-	-	160.0±22.3	-	470
Kelany et al. [44]							471
Control	-	191.1±11.9	-	-	330.1±16.7	-	472
Curcumin 200 mg/kg	-	136.6±7.2	-	-	221.8±2.2	-	473
Kobori et al. [45]							474
Control	4.5±0.2 g	208.9±9.8	-	-	55.2±4.2	-	475
Curcumin 0.1%	4.2±0.2 g	211.4±14.9	-	-	46.1±1.2	-	476
Koboziew et al. [46]							477
Control	25.0±2.0%	-	-	-	-	-	478
Curcumin 0.7%	27.9±3.5%	-	-	-	-	-	479
Lee et al. [47]							480
Control	-	-	-	-	-	2.5±2.0	481
Curcumin	-	-	-	-	-	2.3±1.5	482
Curcumin	-	-	-	-	-	0.8±2.0	483

Li et al. [48]							484
Control	-	11.1±3.0 mg/g	-	-	1.8±6.0 mM/L	-	485
Curcumin 2000 mg/kg	-	6.1±4.5 mg/g	-	-	1.1±1.0 mM/L	-	486
Li et al. [50]							487
Control	-	5.0±0.7 mM/L	0.9±0.1 mM/L	3.5±0.3 mM/L	0.4±0.1 mM/L	-	488
Curcumin 40 mg/kg	-	4.8±0.7 mM/L	0.7±0.1 mM/L	3.8±0.3 mM/L	0.4±0.1 mM/L	-	489
Curcumin 80 mg/kg	-	4.6±0.8 mM/L	0.7±0.1 mM/L	3.8±0.3 mM/L	0.4±0.1 mM/L	-	490
Lovastatin	-	4.5±0.7 mM/L	0.6±0.1 mM/L	3.8±0.5 mM/L	0.4±0.1 mM/L	-	491
Li et al. [51]							492
Control	-	11.8±2.1 mM/L	12.0±2.6 mM/L	0.3±0.2 mM/L	2.4±0.3 mM/L	-	493
Curcumin 100 mg/kg	-	4.2±1.1 mM/L	4.6±0.6 mM/L	1.1±0.1 mM/L	1.2±0.2 mM/L	-	494
Majithiya et al. [52]							495
Control	-	289.4±18.2	-	-	256.2±24.8	-	496
Curcumin 100 mg/kg	-	271.5±22.9	-	-	338.3±19.4	-	497
Curcumin 200 mg/kg	-	204.3±27.6	-	-	296.4±28.7	-	498
Curcumin 400 mg/kg	-	146.7±24.9	-	-	209.5±26.3	-	499
							500
							501
Miyazawa et al. [53]							502
Control	43.2%	-	-	-	-	-	503
Curcumin 1 g/kg	43.8%	-	-	-	-	-	504
Piperine	44%	-	-	-	-	-	505
Neyrinck et al. [55]							506
Control	1.1±0.1 g	5.2±2.5 Mm	-	-	0.2±3.5 mM	-	507
Curcumin 0.3%	1.2±0.0 g	5.2±2.5 mM	-	-	0.2±3.5 mM	-	508
0.1% Berberin	1.1±0.0 g	5.7±2.0 mM	-	-	0.2±3.0 mM	-	509

Pan et al. [57]							
Control	6.6±0.4	-	-	-	6.33±3.5 mg/g	-	
THC 20 mg/kg	5.3±0.8	-	-	-	4.5±0.5 mg/g	-	
THC 100 mg/kg	5.6±0.7	-	-	-	4.0±2.0 mg/g	-	
Preez et al. [58]							
Control (HFD)	184.0±10.0 g	1.5±0.1 mM/L	-	-	1.7±0.5 mM/L	-	
Curcumin 5 mg/kg	185.0±14.0 g	1.7±0.1 mM/L	-	-	1.8±0.1 mM/L	-	
Curcumin 100 mg/kg	202.0±13.0 g	1.5±0.1 mM/L	-	-	1.5±0.1 mM/L	-	
HFD + CCNP	581.0±39.0 g	1.9±0.1 mM/L	-	-	1.6±0.2 mM/L	-	
CCNP	467.0±22.0 g	1.7±0.1 mM/L	-	-	1.5±0.2 mM/L	-	
Ramesh et al. [59]							
Control (HFD)	-	74.8±1.5	31.2±1.1	24.3±1.0	143.3±2.1	-	
Curcumin 50 mg/kg	-	46.8±3.1	21.3±0.8	34.5±1.0	82.8±1.6	-	
Control (HFD + Olanzapine)	-	85.2±1.5	36.0±0.8	15.3±1.0	171.7±3.2	-	
Curcumin 50 mg/kg	-	68.2±1.2	21.7±1.0	24.3±1.1	103.8±1.3	-	
Rao et al. [60]							
Control	-	376.7±0.1	-	-	-	-	
Curcumin 0.1%	-	124.8±0.1	-	-	-	-	

Curcumin 0.25%	-	121.9±0.1	-	-	-	-	530
Curcumin 0.5%	-	128.1±0.1	-	-	-	-	531
Rivego-Sagado et al. [61]							532
Control (Normal)	-	50.0±2.0 mg/mL	-	-	73.3±1.0 mg/g	-	533
Curcumin	-	55.0±2.0 mg/mL	-	-	73.3±1.0 mg/g	-	534
Control (Small)	-	53.3±1.5 mg/mL	-	-	173.3±2.0 mg/g	-	535
Curcumin	-	58.3±1.5 mg/mL	-	-	253.3±2.5 mg/g	-	536
Sarker et al., [63]							537
Control	-	259.1±11.2	153.6±9.6	54.4±2.5	212.0±12.6	-	538
Curcumin 1%	-	181.7±10.4	94.0±4.0	57.3±6.4	190.0±17.3	-	539
Curcumin 2%	-	176.0±9.6	98.0±5.6	61.7±1.5	166.7±7.6	-	540
Curcumin 3%	-	201.7±7.6	110.0±7.0	65.3±1.5	186.0±10.0	-	541
Severcan et al. [64]							542
Control	-	72.3	16.7	27.5	142.8	-	543
Curcumin 100 mg/kg	-	79.9	14.9	24.6	175.9	-	544
Curcumin 200 mg/kg	-	68.8	17.1	26.9	121.6	-	545
							546
Su et al., [65]							547
Control	-	6.6±6.0	4.1±3.9	1.5±0.4	-	-	548
Curcumin 250 mg/kg	-	6.4±10.3	3.0±6.2	1.3±0.7	-	-	549
Tiwari-Pandey et al. [66]							550
Control	0.02	-	-	-	-	-	551
Curcumin 25 mg/kg	0.01	-	-	-	-	-	552
Wu et al. [67]							553
Control	34.0±0.5	-	-	-	-	-	554
Curcumin 2000 mg/kg	31.0±0.5	-	-	-	-	-	555

Zhang et al. [68]						
Control	-	89.35±2.7	36.8±3.1	13.6±1.6	146.0±11.5	-
Curcumin 15 mg/kg	-	70.8±7.0	29.6±1.6	15.9±2.7	115.9±9.7	-
Curcumin 30 mg/kg	-	68.1±7.4	26.9±2.1	17.4±3.1	112.4±11.5	-
Curcumin 60 mg/kg	-	53.8±5.0	22.4±2.2	26.0±2.7	103.5±9.7	-
Pidgitazone	-	60.73±3.5	24.1±1.8	24.0±3.9	102.7±12.4	-
Zou et al. [70]						
Control	-	36.0±3.4	31.6±4.8	6.8±1.5	-	-
Curcumin 1000 mg/kg	-	24.6±6.6	20.0±5.2	9.1±2.0	-	-

Supplementary Table S9. Assessing outcomes related to inflammatory markers, including interleukin-6 (IL-6), interleukin-10 (IL-10), interleukin-12 (IL-12), interleukin-1beta (IL-1β), tumor necrosis factor-alpha (TNF-α:), Interferon-gamma (INF-γ) highlights the effects of kefir and its active components in rodent models of metabolic syndrome.

Author, Year	Inflammatory markers (mean ± SD; pg/mL)					
(tested group)	IL-6	IL-10	IL-12	IL-1β	TNF-α	INF-γ
Afifi et al. [25]						
Control	135.0±0.5	-	-	-	1.5±0.8 ng/mL	-
Curcumin 40 mg/kg	94.0±0.2	-	-	-	0.8±0.1 ng/mL	-
Curcumin 80 mg/kg	88.0±0.2	-	-	-	0.6±0.1 ng/mL	-
Auger et al. [30]						
Control	-	-	-	-	5.2±4.9	-
Curcumin	-	-	-	-	3.5±2.8	-
D' Antongiovanni et al. [32]						
Control	-	-	-	13.3±2.0	-	-
Curcumin 49 mg/kg	-	-	-	5.8±0.5	-	-
Kelany et al. [44]						
Control	-	-	-	-	57.2±1.2	-
Curcumin 200 mg/kg	-	-	-	-	31.0±1.2	-
Kobori et al. [45]						
Control	-	-	-	-	12.8±6.0	48.6±26.3
Curcumin 0.1%	-	-	-	-	6.1±1.1	18.9±11.0
Li et al. [50]						
Control	-	-	-	-	11.4±2.6 U/mL	-
Curcumin 100 mg/kg	-	-	-	-	3.5±0.5 U/mL	-
Miyazawa et al. [53]						
Control	188.5±0.5	90.4	609.3±3.0	13.5±4.5	-	5.6±0.5

Curcumin 1 g/kg	69.2±1.5	42.3	220.4±2.5	12.9±3.5	-	2.8±0.5	593
Mohammadi et al. [54]							594
Control	0.8±2.0	-	-	-	-	-	595
Curcumin 100 mg/kg	0.7±1.7	-	-	-	-	-	596
Curcumin 300 mg/kg	0.6±1.8	-	-	-	-	-	597
Neyrinck et al. [55]							598
Control	1.0±0.1	-	-	-	1.0±0.1	-	599
Curcumin 0.3%	1.8±0.4	-	-	-	2.4±0.6	-	600
0.1% Berberine	1.7±0.2	-	-	-	1.9±0.4	-	601
Omaima and Fouad, [56]							602
Curcumin 200 mg/kg							603
Pre-treatment	43.9±11.6	-	-	2.1±0.5	3.6±0.8	-	604
Post-treatment	24.0±2.3	-	-	0.6±0.4	6.4±1.9	-	605
Irbesartan	28.5±1.9	-	-	1.70.4	3.8±0.7	-	606
Severcan et al. [64]							607
Control	-	-	-	-	61.4	-	608
Curcumin 100 mg/kg	-	-	-	-	36.8	-	609
Curcumin 200 mg/kg	-	-	-	-	32.4	-	610
Su et al. [65]							611
Control	-	-	-	-	79.5±11.2	-	612
Curcumin 250 mg/kg	-	-	-	-	74.8±8.3	-	613
Wu et al. [67]							614
Control	17.5±8.0	-	-	-	-	-	615
Curcumin 2000 mg/kg	10.5±4.5	-	-	-	-	-	616

617

Supplementary Table S10. Assessing outcomes related to oxidative stress markers highlights the effects of curcumin/*Curcuma longa* extracts consumption in rodent models of metabolic syndrome. 618 619

Author, Year	Oxidative stress markers (mean ± SD)						
(tested group)	MDA	GSH	GSR	CAT	TOS	TAC	SOD
Afifi et al. [25]							
Control	5.3±1.5 µM/L	-	-	-	-	-	4.1±0.2 U/mL
Curcumin 40 mg/kg	3.3±1.5 µM/L	-	-	-	-	-	8.2±0.2 U/mL
Curcumin 80 mg/kg	2.6±1.0 µM/L	-	-	-	-	-	8.8±1.0 U/mL
Akintunde et al. [27]							
Control	0.2±3.5 U/g	-	-	204.4±1.5 U/g	-	-	28.9±2.0 U/mg
Curcumin 50 mg/kg	0.1±1.0 U/g	-	-	261.7±2.5 U/g	-	-	38.3V1.5 U/mg
Curcumin 100 mg/kg	0.1±0.5 U/g	-	-	269.6±1.5 U/g	-	-	40.0±1.0 U/mg
Bulboacă et al. [31]							
Control (Fructose)	13.5±3.5 pM/L	-	-	-	70.0 µM/L	0.6 Eq/L	-
Curcumin 1 g/kg	6.0±1.5 pM/L	-	-	-	27.0 µM/L	1.1 Eq/L	-
Demir [33]							
Control	74.4±0.5 nM/g	42.2±0.5 µM/g	42.2±0.5 µg/g	625.0 µg/g	-	-	-
Curcumin 1 mg/kg	51.1±0.5 nM/g	57.8±0.5 µM/g	51.1±0.5 µg/g	833.3 µg/g	-	-	-
Eissa et al. [36]							
Control	40.2±0.3	-	-	-	-	-	-
Curcumin 200 mg/kg	33.5±0.4	-	-	-	-	-	-

Atorvastatin	31.5±0.4	-	-	-	-	-	-	640
Hussein et al. [41]								641
Control	2.5±3.0 μM/mL	-	-	-	-	-	-	642
Curcumin 80 mg/kg	1.1±1.0 μM/mL	-	-	-	-	-	-	643
Kelany et al. [44]								644
Control	2.3±0.8 nM/mL	-	-	-	-	-	-	645
Curcumin 200 mg/kg	1.6±0.1 nM/mL	-	-	-	-	-	-	646
Kobori et al. [45]								647
Control	3.4±0.5 nM/mL	-	-	-	-	-	-	648
Curcumin 0.1%	0.7±0.2 nM/mL	-	-	-	-	-	-	649
Severcan et al. [64]								650
Control	-	-	-	-	14.6 μM/L	1.0 μM/L	-	651
Curcumin 100 mg/kg	-	-	-	-	6.1 μM/L	1.0 μM/L	-	652
Curcumin 200 mg/kg	-	-	-	-	8.1 μM/L	1.0 μM/L	-	653

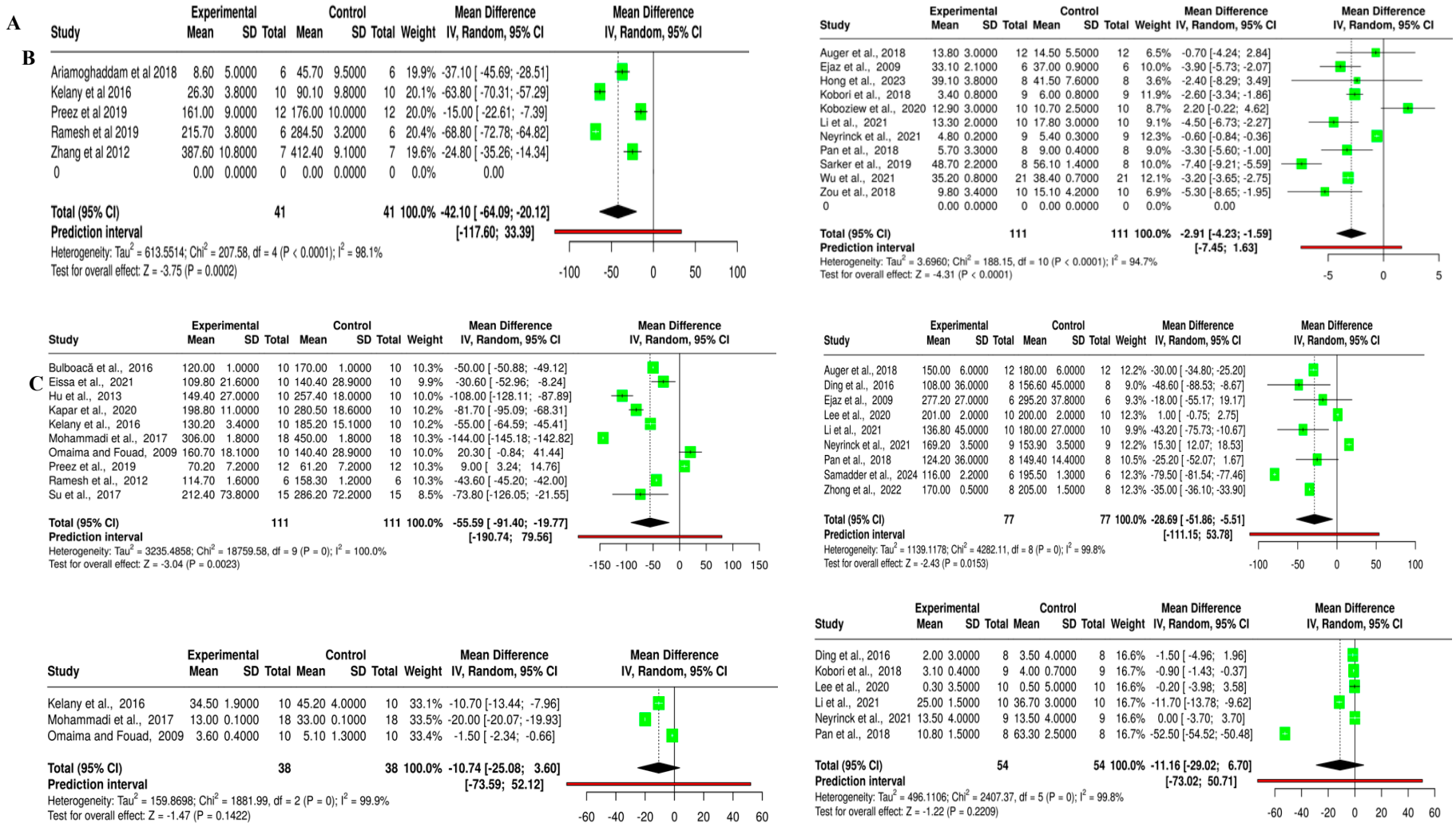

Supplementary Figure S1. Forest plot analysis showing the effects of consuming curcumin/*Curcuma longa extracts* compared to the control group on weight gain (A), glucose level (B), and insulin level (C) in rat (left panel) and mouse (right panel) models.

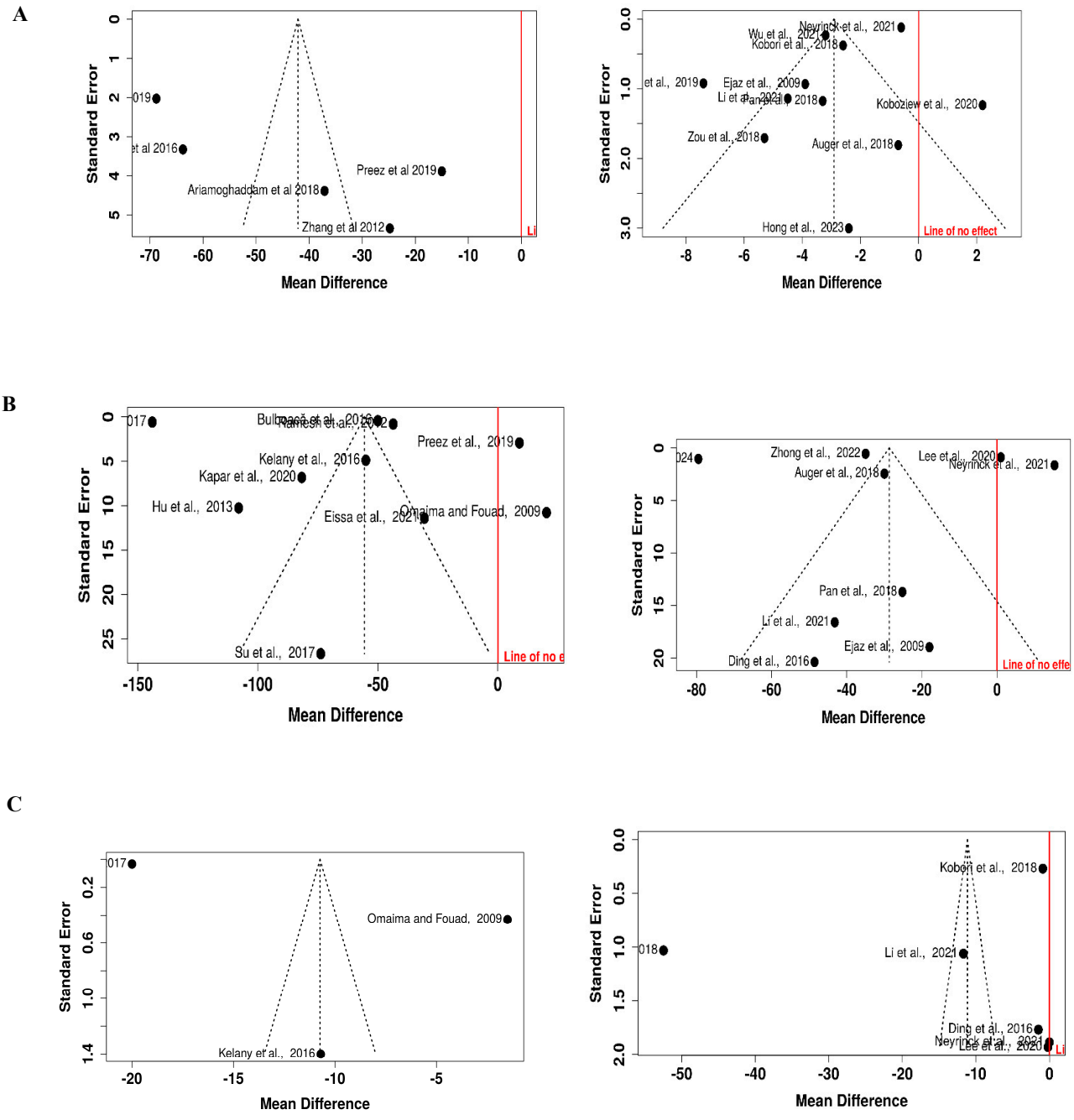

Supplementary Figure S2. A funnel plot illustrating the distribution of publication biases associated with the consumption of curcumin/*Curcuma longa* extracts on weight gain (A), glucose levels (B), and insulin levels (C) in rat (left panel) and mouse (right panel) models.

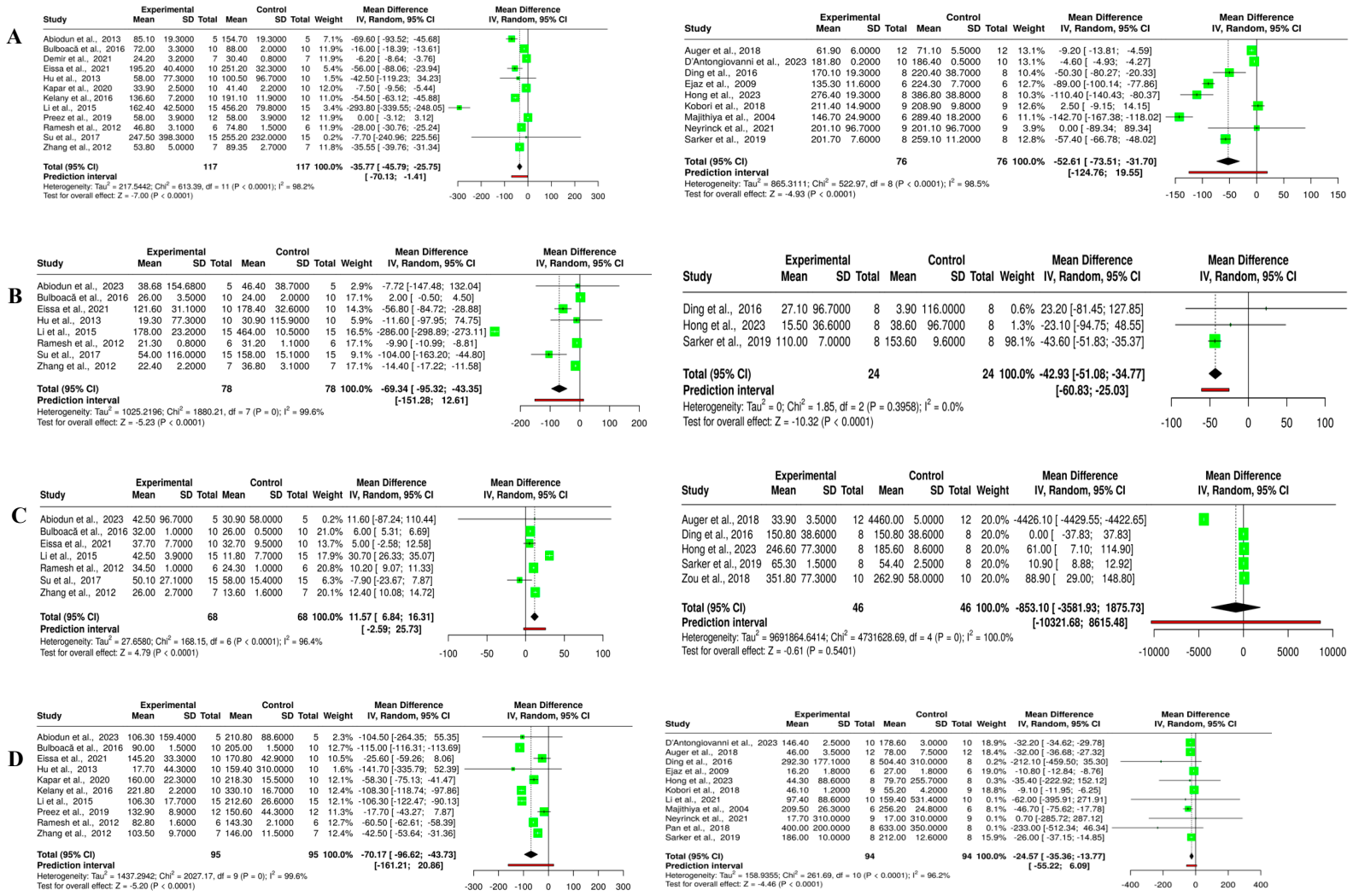

Supplementary Figure S3. A forest plot illustrating the distribution of publication biases associated with the consumption of curcumin/*Curcuma longa extracts* on total cholesterol (A), low-density lipoprotein cholesterol (B), high-density lipoprotein cholesterol (C), and triglyceride (D) levels in the rat (left panel) and mouse (right panel) models.

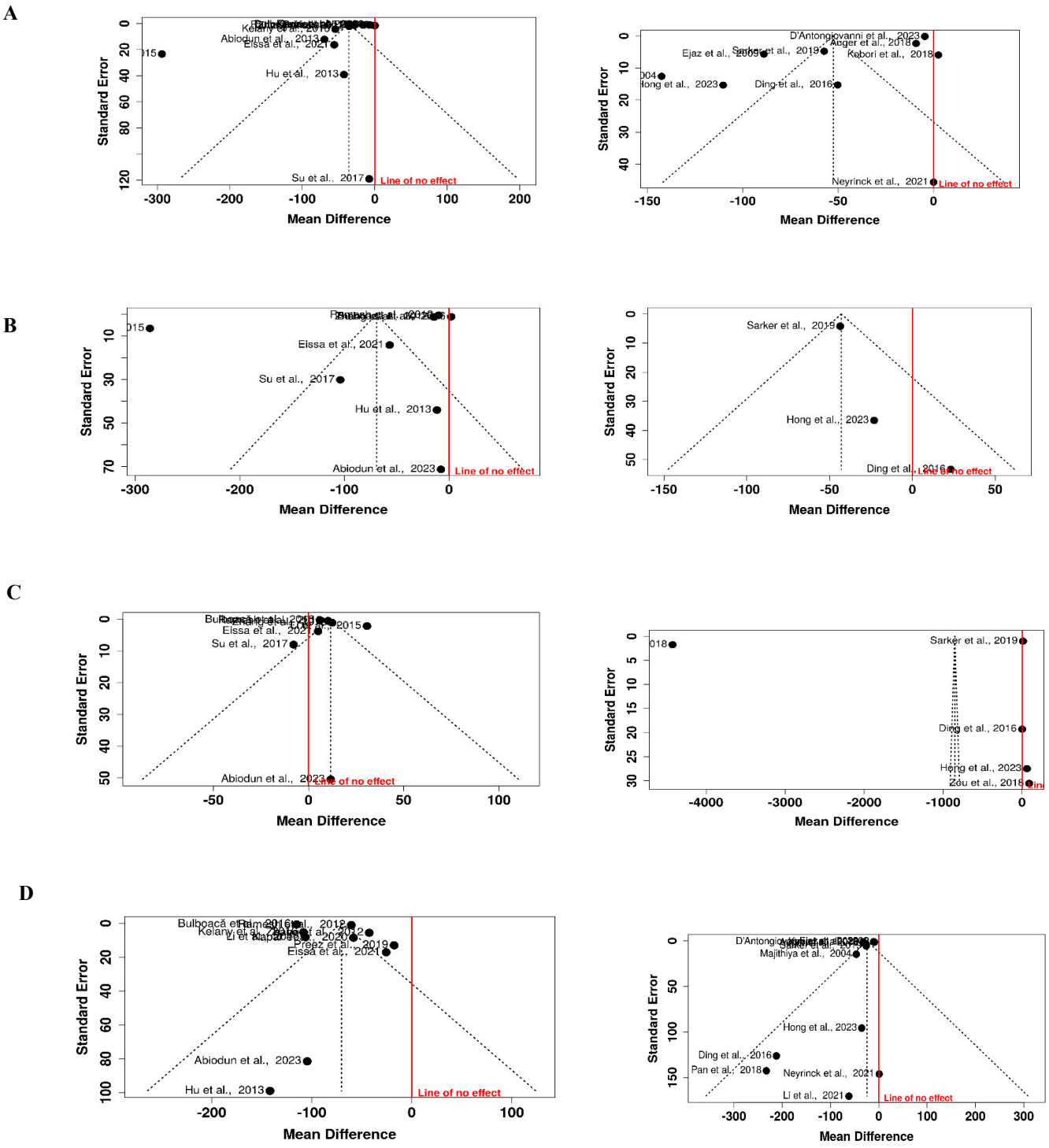

Supplementary Figure S4. A funnel plot illustrating the distribution of publication biases associated with the consumption of curcumin/*Curcuma longa extracts* on total cholesterol (A), low-density lipoprotein cholesterol (B), high-density lipoprotein cholesterol (C), and triglyceride (D) levels in the rat (left panel) and mouse (right panel) models.
